# A Compartmentalized Joint‐on‐chip (JoC) Model to Unravel the Contribution of Cartilage and Synovium to Osteoarthritis Pathogenesis

**DOI:** 10.1002/advs.202500374

**Published:** 2025-09-11

**Authors:** Cecilia Palma, Shima Salehi, Michela Anna Polidoro, Matteo Moretti, Marco Rasponi, Silvia Lopa, Paola Occhetta

**Affiliations:** ^1^ Department of Electronics Information and Bioengineering Politecnico di Milano Via Ponzio 34/5 Milan 20133 Italy; ^2^ Cell and Tissue Engineering Laboratory IRCCS Istituto Ortopedico Galeazzi Via Cristina Belgioioso 173 Milan 20157 Italy; ^3^ Hepatobiliary Immunopathology Laboratory IRCCS Humanitas Research Hospital Via Alessandro Manzoni, 56 Rozzano Milan 20089 Italy; ^4^ Regenerative Medicine Division, Institute for Translational Research Ente Ospedaliero Cantonale – Università della Svizzera Italiana Via Chiesa 5 Bellinzona 6500 Switzerland; ^5^ Euler Institute, Faculty of Biomedical Sciences Università della Svizzera Italiana (USI) Via Buffi 13 Lugano 6900 Switzerland; ^6^ BiomimX Srl Viale Decumano 41, MIND – Milano Innovation District Milan 20157 Italy

**Keywords:** cartilage, inflammation, joint‐on‐chip, mechanical stimulation, organ‐on‐chip, Osteoarthritis, synovium

## Abstract

Osteoarthritis (OA) is a joint disorder causing pain and disability, yet effective treatments are limited due to incomplete understanding of pathogenic mechanisms involving complex tissue interactions. Articular cartilage degradation is a hallmark, resulting from an imbalance in extracellular matrix turnover, influenced by mechanical and biochemical signals. The synovium also plays a central role in joint inflammation, with macrophages and fibroblasts releasing pro‐inflammatory cytokines and degradative enzymes. However, understanding cartilage‐synovium interactions in OA pathogenesis remains challenging. Here, a compartmentalized joint‐on‐chip (JoC) model that enables independent culture of 3D human cartilage and synovium constructs, allowing spatio‐temporal control over their communication, is presented. The JoC platform supports induction of OA characteristics in both tissues, by applying hyper‐physiological compression to cartilage constructs to mimic mechanical damage and by treating synovium constructs with TNFα and IFNγ to simulate inflammation. Moreover, the platform enables exploration of paracrine signaling between these tissues under pathophysiological conditions, showing that inflamed synovium constructs induce early cartilage degradation, while mechanically damaged cartilage promotes macrophage activation and inflammatory responses in the synovium. These findings support a bidirectional relationship in OA onset and underscore the JoC model as a tool for studying joint tissue interactions.

## Graphical abstract

A compartmentalized joint‐on‐chip (JoC) platform is here developed, modelling the interactions between cartilage and synovium in osteoarthritis (OA). Using independent culture and mechanical/biochemical stimulation, the JoC revealed how inflammation and mechanical damage drive mutual tissue changes. This tool offers new insights into OA onset and potential therapeutic targets by replicating complex joint dynamics in a controlled environment.

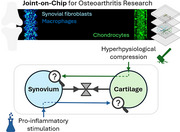

## 1 Introduction

Osteoarthritis (OA) is the most common degenerative joint disorder that affects diarthrodial joints and a major cause of disability in the aging population.^[^ ^1^, ^2^, ^3^ ^]^ Despite its high prevalence, current therapies predominantly offer palliative relief and fall short of re‐establishing a physiological state in the joint.^[^ ^4^ ^]^ This limitation stems from an incomplete understanding of the initial mechanisms of the disease, attributed to its complexity and multifactorial nature.^[^ ^5^, ^6^ ^]^ Indeed, OA leads to a systemic failure of the entire synovial joint, with alterations in multiple components, including articular cartilage and synovium.^[^ ^7^ ^]^ Cytokines and growth factors play a pivotal role in initiating cartilage matrix breakdown and eliciting abnormal cellular responses,^[^ ^8^, ^9^ ^]^ leading to disruption of the collagen network, depletion of proteoglycans, as well as inappropriate hypertrophic‐like maturation and cartilage calcification.^[^ ^10^, ^11^, ^12^, ^13^ ^]^ Moreover, mechanical factors, including obesity, trauma, or joint misalignment, are strongly associated with OA onset in articular cartilage, classifying the biomechanical environment as a crucial factor in the progression of the disease.^[^ ^14^ ^]^ Concurrently, synovial inflammation, namely synovitis, and the resultant release of pro‐inflammatory mediators are also considered as key players in the pathogenesis of OA.^[^ ^15^ ^]^ During OA, synovial resident macrophages and synovial fibroblasts (SFBs) release a variety of pro‐inflammatory mediators.^[^ ^16^ ^]^ This occurs together with the infiltration of inflammatory cells within the synovial membrane, predominantly macrophages and T cells.^[^ ^17^ ^]^ Such inflammatory milieu exacerbates synovial inflammation and further drives cartilage degradation, leading to the release of degradative enzymes and pain‐related factors.^[^ ^18^, ^19^, ^20^ ^]^ While both cartilage degradation and synovitis are well‐established hallmarks of OA progression, the causal relationship between these tissues remains unclear.

In this context, Organs‐on‐Chip (OoC) can be used to unravel joint tissues interactions during OA and disentangle the cause‐and‐effect relationships between the various factors involved in the disease development. Nevertheless, the first pioneering microphysiological models focused on the physiopathology of chondrocytes alone.^[^ ^21^, ^22^, ^23^, ^24^, ^25^, ^26^, ^27^, ^28^ ^]^ Among others, we have previously developed a cartilage‐on‐chip model by culturing human articular chondrocytes in 3D within a mechanically active OoC platform.^[^ ^29^ ^]^ This study demonstrated that OA traits, e.g., catabolism and hypertrophy, could be induced through the sole application of a 30% hyperphysiological compression (HPC), confirming the leading role of mechanical alteration in the disease pathogenesis. However, to better capture OA as a whole‐joint disease, different cellular components should be included into a joint‐on‐chip model, including cartilage, subchondral bone,^[^ ^30^, ^31^, ^32^ ^]^ and synovial membrane. Among others, several microfluidic platforms have recently emerged to specifically investigate cartilage‐synovium interactions.^[^ ^33^ ^]^ Petta et al.^[^ ^34^ ^]^ presented a patient‐specific joint‐on‐chip model culturing SFBs and chondrocytes in separate compartments exposed to OA synovial fluid, enabling drug screening and inflammatory response profiling. In another study from Li et al.^[^ ^35^ ^]^ a human mesenchymal stem cell‐derived miniaturized joint system (i.e., miniJoint) was developed, incorporating osteochondral complex, synovial‐like fibrous tissue, and adipose tissue, to recapitulate synovitis and assess its impact on other tissues. While such models showed considerable potential for translational research and yielded valuable insights for evaluating therapeutics,^[^ ^34^, ^36^ ^]^ they present common limitations, such as absence of an immune component and the inability to resolve tissue‐specific directional effects, thereby limiting the mechanistic dissection of cartilage–synovium interactions.

Mirazi et al.^[^ ^37^ ^]^ introduced a microfluidic co‐culture platform incorporating four joint‐relevant human primary cell types including immune cells (i.e., osteoblasts, chondrocytes, fibroblasts, and M0/M1 macrophages), to mimic paracrine interactions in healthy and inflammatory joint environments. However, the model focused primarily on cytotoxicity and metabolic assays, without investigating tissue‐specific molecular and phenotypic responses. In a previous work reported by our research group, Mondadori et al.^[^ ^38^ ^]^ developed a microfluidic immunocompetent organotypic model, characterized by a synovial compartment, a perfusable endothelialized channel, a compartment for synovial fluid, and a cartilage compartment. The platform was exploited to assess monocyte extravasation from the endothelial channel to the synovium in response to exposure to OA synovial fluid. However, the potential detrimental impact exerted by extravasated monocytes on the cartilage compartment was not further investigated.^[^ ^36^ ^]^ Of note, none of the abovementioned studies investigated the role of a mechanically active environment in perturbating cartilage‐synovium cross‐talk.

Here, we present a compartmentalized joint‐on‐chip (JoC) model enabling the independent generation and selective perturbation of 3D human cartilage and synovial micro‐constructs, to investigate how the unbalanced communication between these tissues contributes to the development of OA. This platform allows spatiotemporal control of inter‐tissue communication and the targeted induction of OA‐like traits in either compartment, thereby supporting precise investigation of cause–effect relationships. Specifically, the JoC model was employed here to assess whether mechanically damaged cartilage triggers inflammatory changes in healthy synovium, and conversely, whether an inflamed synovium induces cartilage degradation.

## 2 Results

### 2.1 Device concept

**Figure 1:**
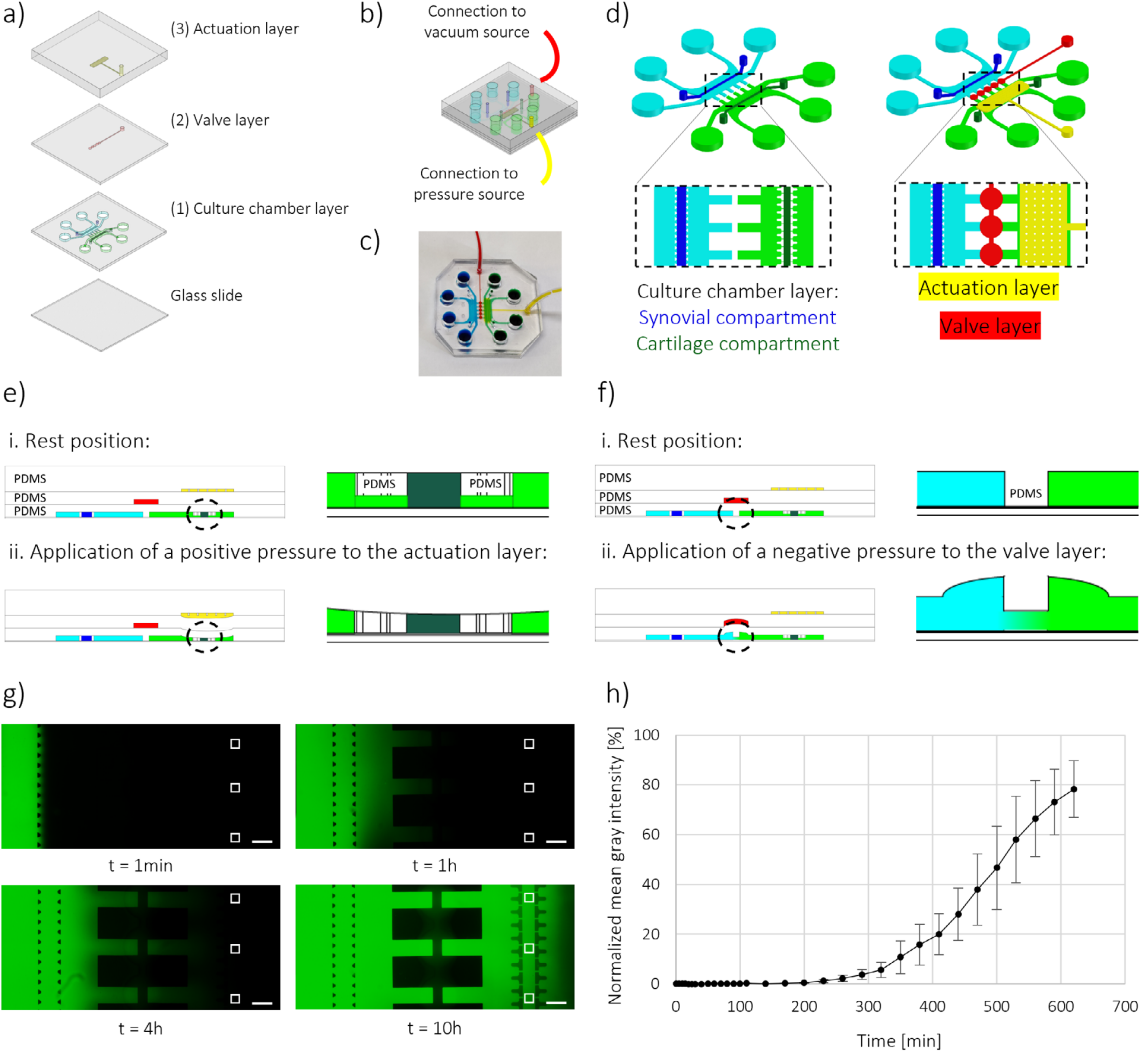
Device concept. a) 3D representation of the PDMS layers composing the microfluidic platform, aligned on top of a glass slide, i.e., the bottom culture chamber layer (1), the middle valve layer (2), and the top actuation layer (3). b) 3D representation of the assembled device. The actuation layer is connected to a pressure source, while the valve layer is connected to a vacuum source. c) Photograph of an assembled device, in which different color dyes were used to identify the compartments: blue for the synovial compartment, green for cartilage compartment, red for the valve layer, and yellow for the actuation layer. d) Schematic representation of the device (3D and top view), highlighting culture chamber layer with synovial and cartilage compartments, actuation layer and valve layer. e) Side view showing actuation working mechanisms: at rest (i), cartilage tissue is not mechanically stimulated. Upon pressurization of the actuation layer (ii), cartilage micro‐constructs are subjected to confined compression. f) Side view showing valve working mechanisms: at rest (i), the two culture compartments are fluidically independent. Upon vacuum application to the valve layer (ii), PDMS gates between the valves are lifted up and the two culture compartments are put in communication. g) Fluorescence images showing FITC‐dextran diffusion over time. Representative time‐points were chosen. White squares indicate the ROIs where gray intensity was analyzed. Scale bar 500 µm. h) Plot of FITC‐dextran normalized mean fluorescence intensity in the cartilage compartment, for different time‐points.

A JoC device was designed to allow the co‐culture of cartilage and synovium tissues and the selective induction of OA traits. The design builds upon the platform presented by Occhetta et al.,^[^ ^29^ ^]^ with several improvements. Three main requirements were considered when designing the JoC platform: i) the presence of two compartments for the co‐culture of cartilage and synovium with different culture timings, ii) the possibility to independently perturb the compartments either biochemically or mechanically, and finally iii) the ability to evaluate the effect of paracrine communication between the two compartments, both spatially and temporally. Thus, the JoC device is composed of three PDMS layers, assembled on top of a glass slide (**Figure** **1**a): 1) a bottom *culture chamber layer*, 2) a middle *valve layer*, and 3) a top *mechanical actuation layer*. An assembled device is depicted in Figure 1b, together with a representative picture (Figure 1c). The *culture chamber layer* consists of two separate culture compartments for synovium and cartilage cultures, respectively (Figure 1d, Figure S1a). Both culture compartments are composed of a central channel to host 3D micro‐constructs, flanked by two lateral medium channels. The *synovial compartment* includes a 400 µm‐wide central channel, limited by two rows of trapezoidal pass‐through posts, while the *cartilage compartment* includes a 300 µm‐wide central channel, limited by two rows of T‐shaped overhanging posts, which serve a dual purpose: confining cell‐laden hydrogel and allowing the application of a 30% mechanical compression to cartilage micro‐constructs, as described by Occhetta et al.^[^ ^29^ ^]^ A gap separates the hanging posts from the underlying glass slide at rest. Upon pressurization of the actuation compartment (Figure 1e), the ceiling of the chamber bends downward until the bases of the posts abut against the glass slide, causing confined compression of the cartilage micro‐constructs. This 43 µm gap defines the mechanical strain, enabling the application of 30% HPC to the cartilage constructs.^[^ ^39^ ^]^ Finally, the two culture compartments, which are independent at rest, can be interconnected through the integration of normally closed (NC) curtain valves,^[^ ^40^, ^41^, ^42^, ^43^ ^]^ here named *communication valves* (Figure 1f). When at rest, PDMS gates located between the two culture compartments completely lay on the underneath glass slide, thus sealing off communication channels and preventing fluid passage between the compartments. Upon *communication valves* opening through the application of negative pressure, PDMS gates are lifted and paracrine communication between the culture compartments is allowed. The valve design enables reversible opening and resealing, preserving compartmental isolation (Video S1). This spatiotemporal control allows independent tissue maturation in tailored culture media and the selective induction of inflammatory traits before initiating inter‐tissue interactions.

### 2.2 Technical characterization of the JoC platform

*Communication valves* in the JoC platform were designed to maintain the two culture compartments fluidically isolated at rest, while allowing communication through molecular diffusion upon vacuum application, as demonstrated in Figure S1b with color dyes. To evaluate the value of negative pressure required to fully open the valves, a calibration was performed. Briefly, the valve layer was filled with a red dye, and pressure was progressively decreased from 0 to −660 mmHg. As the pressure decreased, the PDMS walls deflected, displacing the dye and rendering the area transparent at full valve opening. Valves reached maximum opening at a pressure of ‐580 mmHg, as evidenced by the plateau in the normalized mean gray intensity (MGI) curve in Figure S1c. Importantly, this pressure value is compatible with standard vacuum sources present in biological laboratories (e.g., biological hood aspiration systems).

A functional validation was performed to estimate the kinetics of cytokine diffusion from one culture compartment to the other one upon valve opening, using 40 kDa FITC‐dextran, as analogue for the molecular weight of cytokines.^[^ ^44^ ^]^ First, the central channel of each culture compartment was filled with fibrin gel. FITC‐dextran (1.5 mg mL^−1^) was then injected in the outermost medium channel of the *synovium compartment* (Figure S1d) and FITC‐dextran diffusion toward the *cartilage compartment* was monitored by acquiring images every 10 min for the first 2 h, and then every 30 min up to 10 h. As shown in Figure 1g, FITC‐dextran diffused throughout the entire *synovial chamber* in 1 h and started diffusing toward the *cartilage compartment* after ≈4 h. Finally, after 10 h, FITC‐dextran intensity appeared similar in both compartments. The curve of normalized MGI taken in the *cartilage compartment* over time confirmed these results (Figure 1h). The presence of FITC‐dextran in the gel channel of the *cartilage compartment* could indeed be appreciated starting from 4 h, with a value of 1.27 ± 0.68%. Normalized MGI gradually increased over time, meaning that FITC‐dextran diffusion was continuous and sustained. Finally, after 10 h normalized MGI reached a value of 78.28 ± 11.37%, demonstrating that the two compartments were properly in communication upon valves opening and proving that cytokines could diffuse from one compartment to the other due to a concentration gradient, almost reaching species equilibrium after 10 h.

Finally, based on previous work,^[^ ^29^ ^]^ the actuation layer was designed to provide a HPC to the 3D cartilage micro‐tissues. A mechanical characterization was performed to assess the working actuation pressure of the new system layout and to verify the conformity of the strain field with the values computed through numerical and experimental methods in the previous version of the platform (Figure S2).

### 2.3 Generation of 3D Cartilage Micro‐Tissues Inside the JoC Platform and Induction of an OA Phenotype through Mechanical Overload

The JoC platform was first used to generate healthy cartilage micro‐constructs within the *cartilage compartment*, adapting the protocol developed by Occhetta et al.^[^ ^29^ ^]^ Subsequently, the constructs were induced to acquire an OA phenotype through the application of HPC, to confirm the results obtained in the previous version of the platform.

**Figure 2:**
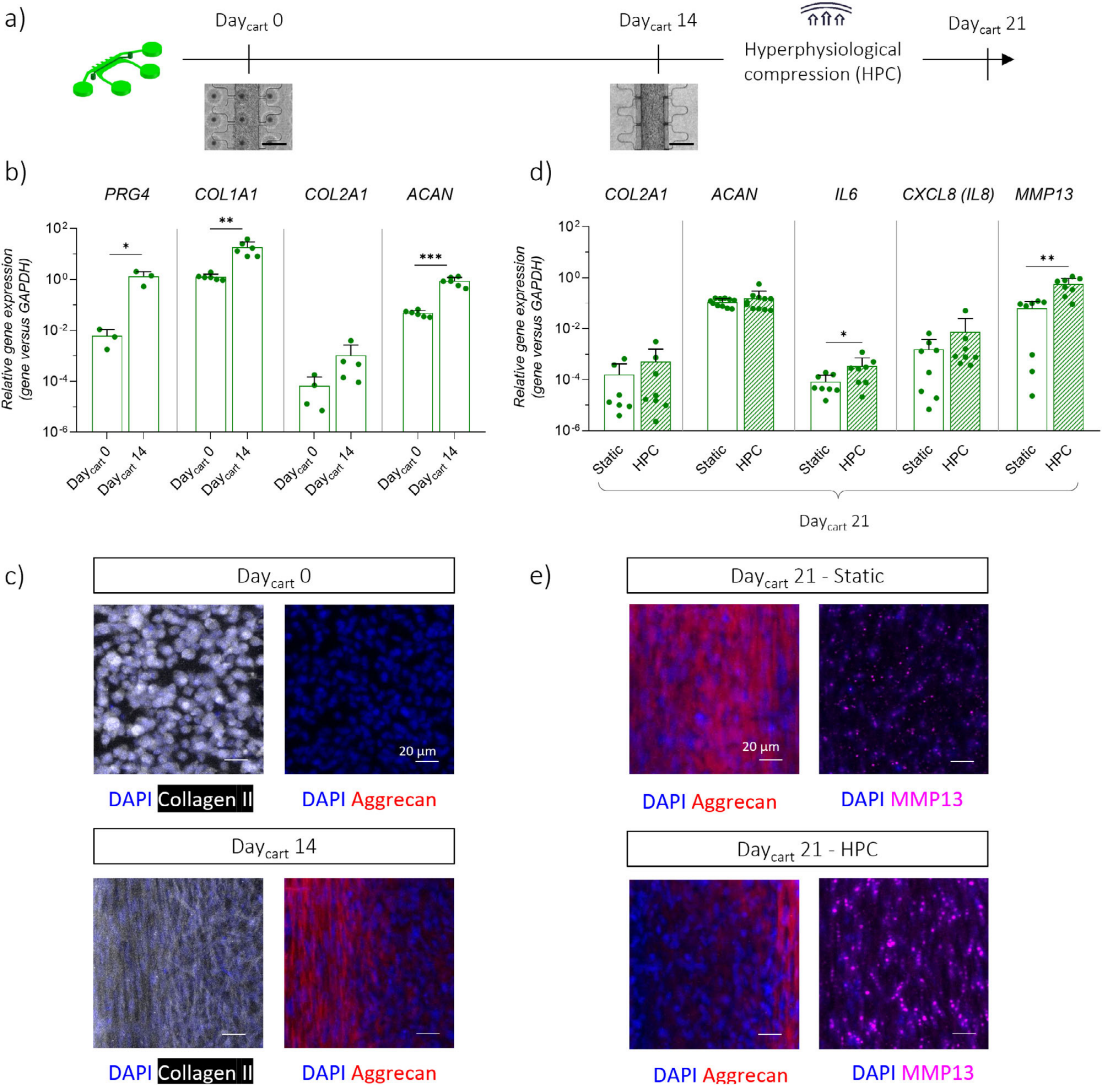
Generation of 3D cartilage micro‐tissues and Induction of an OA phenotype inside the JoC platform: a) Experimental plan, with brightfield images showing cartilage compartment. Scaler bar 300 µm. hACs embedded in a fibrin gel were seeded in the cartilage compartment on Day_cart_ 0 and cultured up to Day_cart_ 14. A cyclic HPC was applied for 7 days to induce OA traits. b) Gene expression analysis of cartilage micro‐constructs. Relative gene expression is shown, i.e., expression of gene of interest normalized against the expression of reference gene (GAPDH). Data are plotted as mean + SD (n ≥ 4); *p < 0.05, **p<0.01, ***p<0.001. c) Confocal images showing the synthesis of collagen‐type II (magenta) and aggrecan (red) inside the cartilage micro‐tissues. Nuclei in blue (DAPI). Scale bar 20 µm. d) Gene expression analysis of cartilage micro‐constructs on Day_cart_ 21, comparing control samples (“Static”) and mechanically stimulated samples (“HPC”). Relative gene expression is shown, i.e., expression of gene of interest normalized against the expression of reference gene (GAPDH). Data are plotted as mean + SD. n ≥ 6, *p < 0.05, **p<0.01. e) Confocal images showing SOX9 (green), aggrecan (red) and MMP13 (magenta) inside the cartilage micro‐tissues, in static versus HPC samples. Scale bar 20 µm.

Primary human articular chondrocytes (hACs) embedded in fibrin gel were cultured under static conditions in the *cartilage compartment* of the JoC platform for 14 days (**Figure** **2**a). As shown in Figure 2b, gene expression analysis demonstrated a significant upregulation of *PRG4* and ECM‐related genes, namely *COL1A1* and *ACAN*, at Day_cart_ 14 compared to Day_cart_ 0. Moreover, an increasing trend was detected for *COL2A1*. The achievement of healthy cartilage micro‐tissues was further proved at protein level as shown by immunofluorescence analysis, revealing a higher deposition of collagen‐type II and aggrecan at Day_cart_ 14 as compared to Day_cart_ 0 (Figure 2c). A 3D reconstruction of the cartilage micro‐tissue cultured within the device is reported in Figure S3a.

After 14 days of cartilage maturation (Day_cart_ 14), the platforms were mechanically actuated with cyclic HPC for one week (Figure 2a). At the gene level, the induction of an OA phenotype was shown by the significant upregulation of the pro‐inflammatory gene *IL6* and the matrix‐degrading enzyme *MMP13* in HPC samples compared to static controls (Figure 2d). Moreover, the pro‐inflammatory gene *CXCL8* (*IL8*) showed an increasing trend. However, no significant differences in *COL2A1* and *ACAN* gene expression were observed. At the protein level, matrix degradation was supported by immunofluorescence staining, which showed increased intracellular MMP13 and decreased aggrecan deposition in HPC samples compared to controls (Figure 2e). Finally, HPC samples exhibited lower levels of SOX9 (Figure S4). All these results were consistent with findings reported by Occhetta et al. in a previously presented single‐compartment platform.^[^ ^29^ ^]^

### 2.4 Generation of 3D Synovial Micro‐Tissues Inside the JoC Platform

A protocol was then established to generate a 3D model of synovium by co‐culturing human monocyte‐derived macrophages (MΦs) and SFBs embedded in fibrin/collagen gel within the *synovium compartment* of the JoC platform for up to 7 days.

**Figure 3:**
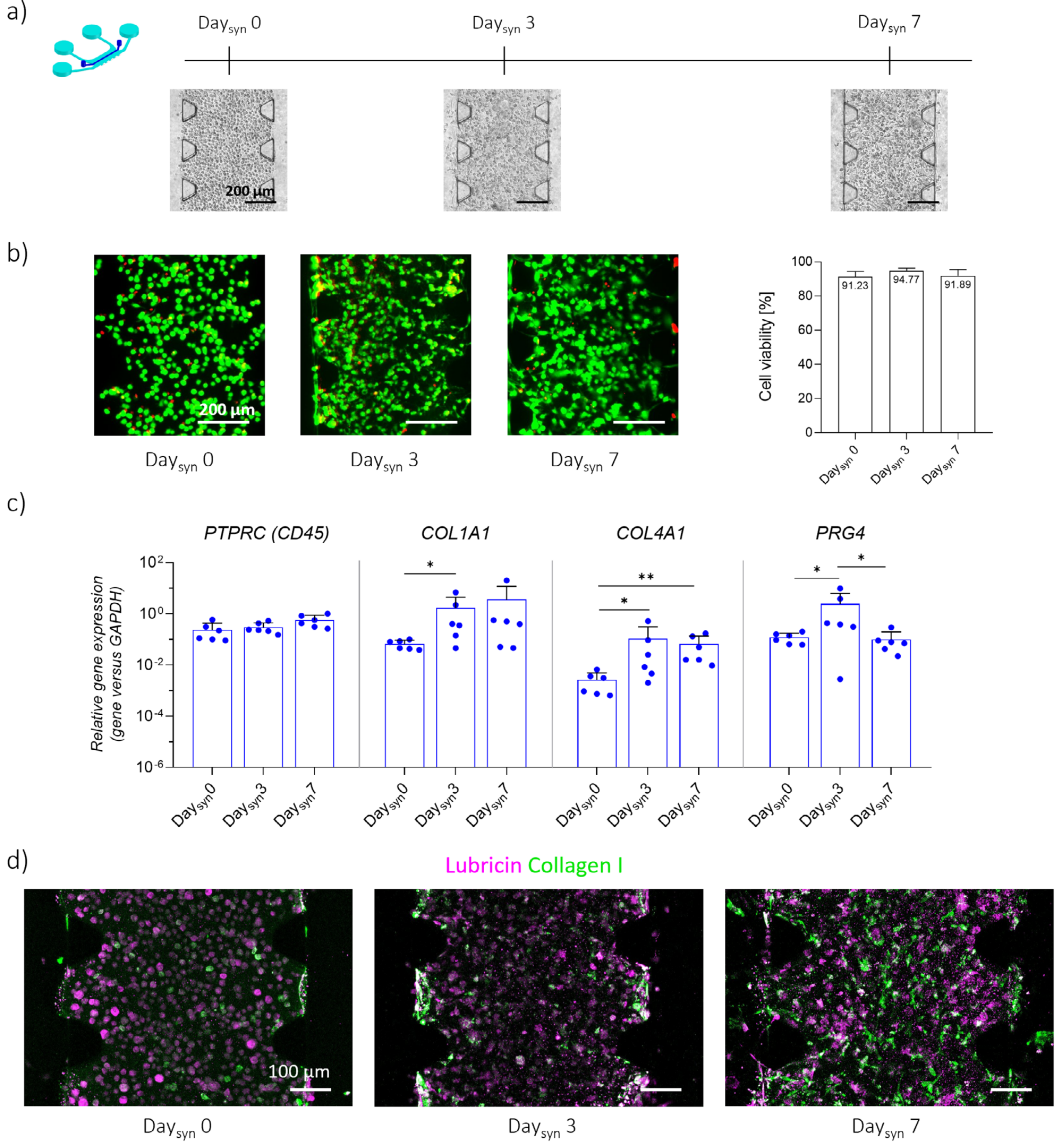
Generation of 3D synovium micro‐tissues inside the JoC platform. a) Experimental plan, with brightfield images showing synovial compartment. Scaler bar 200 µm. MΦs and SFBs embedded in a fibrin/collagen gel were seeded in the synovial compartment on Day_syn_ 0 and cultured up to 7 days. End‐point analyses were conducted at Day_syn_ 0, Day_syn_ 3 and at Day_syn_ 7. b) Live/Dead assay on synovial micro‐constructs on Day_syn_ 0, Day_syn_ 3 and Day_syn_ 7. Alive cells in green, dead cells in red. Scale bar 200 µm. The graph shows cell viability estimated from Live/Dead images (n = 3). Data are plotted as mean + SD. c) Gene expression analysis of synovial micro‐constructs on Day_syn_ 0, Day_syn_ 3 and Day_syn_ 7. Relative gene expression is shown, i.e., expression of gene of interest normalized against the expression of reference gene (GAPDH). Data are plotted as mean ± SD. n = 6, ^*^ *p* < 0.05, ^**^ *p*<0.01. d) Confocal images showing the synthesis of lubricin (magenta) and collagen type‐I (green) inside the synovial micro‐tissues on Day_syn_ 0, Day_syn_ 3 and Day_syn_ 7. Scale bar 20 µm.

As shown in **Figure** **3**b, Live/Dead assays revealed consistently high cell viability (>90%) at all time points: 91.23%, 94.77% and 91.89% at Day_syn_ 0, Day_syn_ 3 and Day_syn_ 7, respectively. At the gene level (Figure 3c), the expression of *PTPRC*, encoding the hematopoietic marker *CD45* and used as an indicator of MΦs survival, remained stable for the whole culture period. Moreover, the expression of *COL1A1* and *COL4A1* showed an increasing trend over time, with significantly higher levels at Day_syn_ 3 with respect to Day_syn_ 0, and a significantly higher expression of *COL4A1* at Day_syn_ 7 with respect to Day_syn_ 0. Finally, the expression of *PRG4*, encoding lubricin, was significantly upregulated at Day_syn_ 3 compared to Day_syn_ 0, but decreased to baseline levels after 7 days. At the protein level (Figure 3c), lubricin was detected in the *synovial compartment* during the whole culture period, with no marked changes among different time‐points. On the other hand, collagen type‐I was barely detectable at Day_syn_ 0 and Day_syn_ 3, while a high presence of collagen‐type I was appreciable at Day_syn_ 7. A 3D reconstruction of the synovium micro‐tissue cultured within the device is reported in Figure S3b. Together, the upregulation of ECM markers and consistent lubricin presence suggested successful maturation of the synovial micro‐constructs.

### 2.5 Induction of Synovial Inflammation in the JoC Platform

**Figure 4:**
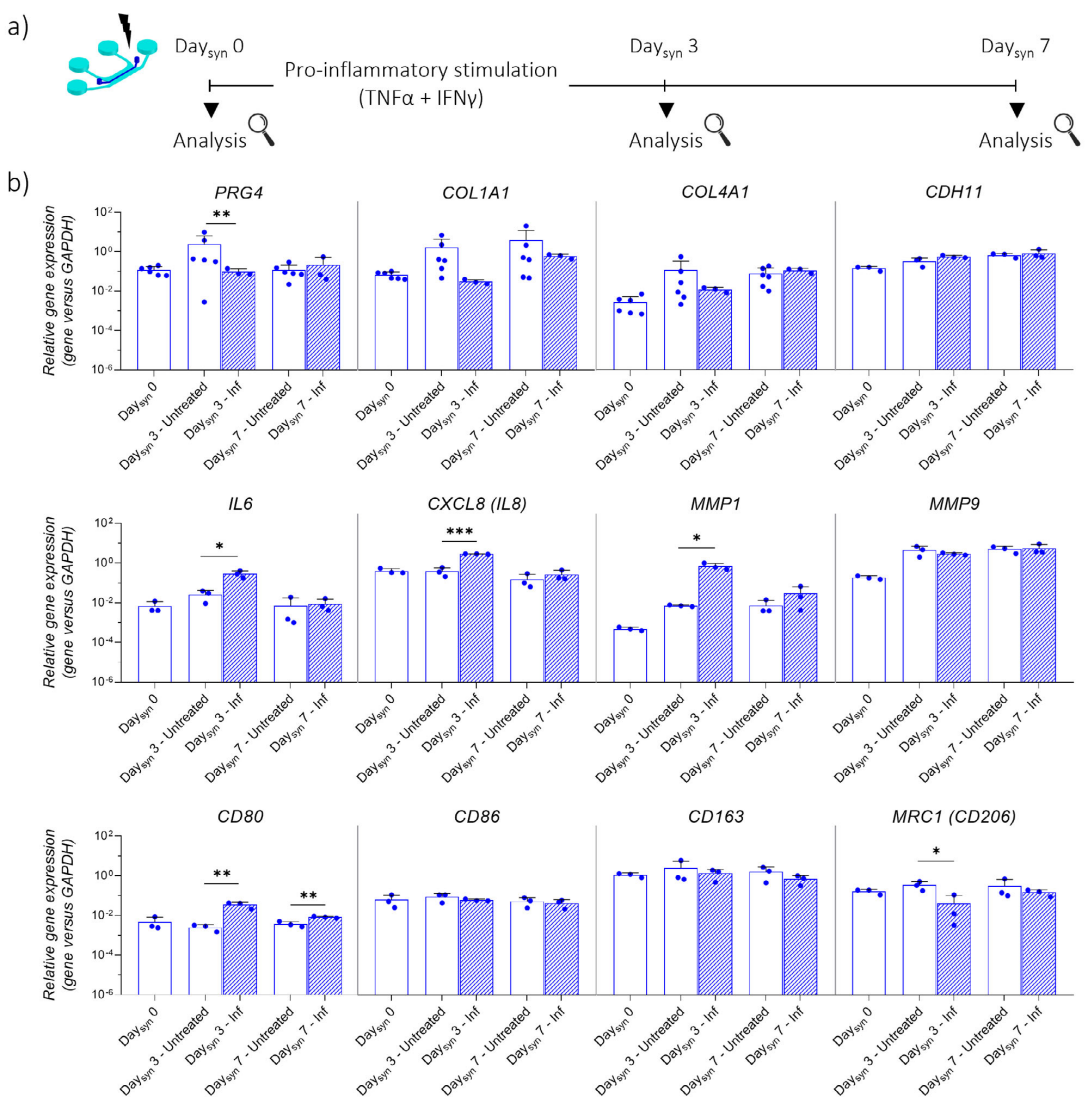
Induction of synovial inflammation in the JoC platform. a) Experimental plan. SFBs and MΦs embedded in fibrin/collagen gel were seeded in the synovium compartment of the JoC platform on Day_syn_ 0. A pro‐inflammatory stimulation (100 ng mL^−1^ IFNγ, 100 ng mL^−1^ TNFα) was supplied up to Day_syn_ 3. From Day_syn_ 3 from Day_syn_ 7, pro‐inflammatory stimuli were removed from the CM to verify whether the pro‐inflammatory stimulus could be maintained stable. End‐point analyses were conducted either at Day_syn_ 0, Day_syn_ 3 and at Day_syn_ 7. b) Gene expression analysis of synovial micro‐constructs on Day_syn_ 0, Day_syn_ 3 and Day_syn_ 7, comparing control samples (“Untreated”) and stimulated samples (‘Inf). Relative gene expression is shown, i.e., expression of gene of interest normalized against the expression of reference gene (GAPDH). Data are plotted as mean + SD. (n = 3) ^*^ *p* < 0.05, ^**^ *p*<0.01, ^***^ *p*<0.001.

To replicate an inflammatory state in the synovial micro‐constructs, macrophage polarization into the proinflammatory M1 state was induced on‐chip, through adaptation of an established protocol.^[^ ^45^ ^]^ Briefly, MΦs and SFBs seeded in the JoC platform were stimulated for three days (i.e., from Day_syn_ 0 to Day_syn_ 3) with an inflammatory cocktail composed of 100 ng mL^−1^ TNFα and 100 ng mL^−1^ IFNγ, followed by four days without stimulation (i.e., from Day_syn_ 3 to Day_syn_ 7, **Figure** **4**a).

At the gene level (Figure 4b), a significant downregulation of *PRG4* was detected at Day_syn_ 3 in inflamed samples compared to untreated controls, but the variation between the two conditions attenuated at Day_syn_ 7, i.e., four days after withdrawal of the pro‐inflammatory stimulation. With respect to ECM‐related genes, *COL1A1* showed a decreasing trend in inflamed samples at Day_syn_ 3, while no differences were observed for *COL4A1*. No relevant differences were observed at Day_syn_ 7. Moreover, pro‐inflammatory stimulation did not induce changes in *CDH11*, which encodes the cell adhesion molecule cadherin‐11, known to regulate SFB function and promote the secretion of proinflammatory factors.^[^ ^46^, ^47^ ^]^ Conversely, treatment with TNFα and IFNγ induced a significant upregulation in pro‐ inflammatory and pro‐degradative genes, i.e., *IL6, CXCL8 (IL8)* and *MMP1*, at Day_syn_ 3, even though this effect leveled off by Day_syn_ 7. In contrast, no significant changes were found for *MMP9* expression in inflamed samples, at any time point.

The expression of macrophage phenotypic markers was then assessed at distinct levels. Gene expression analysis revealed a significant upregulation of the M1 marker *CD80* in inflamed samples compared to untreated controls, both at Day_syn_ 3 and at Day_syn_ 7. No significant changes were observed for *CD86* expression. Furthermore, a significant downregulation of the anti‐inflammatory marker *CD206* was detected at Day_syn_ 3, and a decreasing trend was maintained up to Day_syn_ 7. Similarly, a decreasing trend was observed for the anti‐inflammatory marker *CD163*, at both time points.

**Figure 5:**
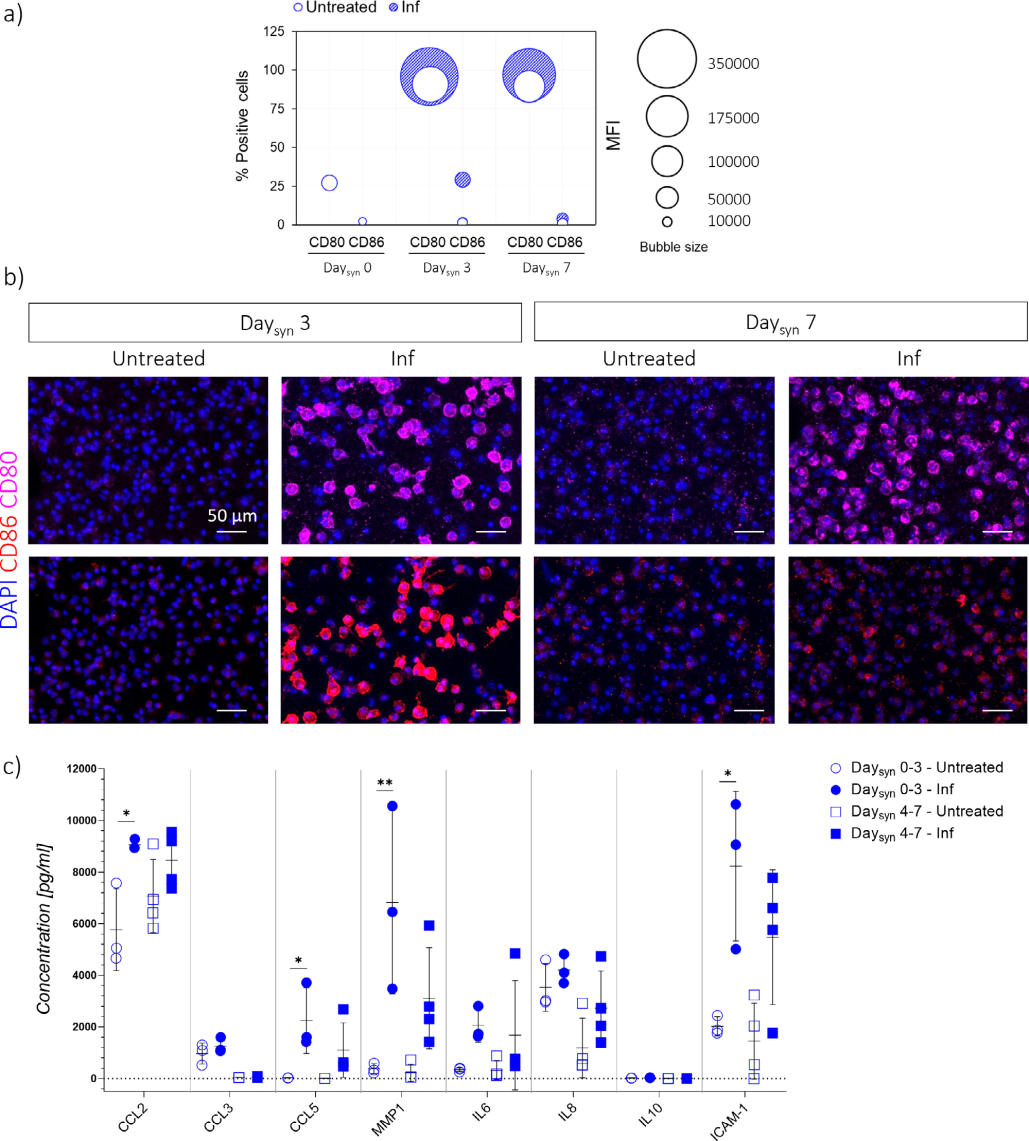
Induction of synovial inflammation in the JoC platform (II). a) Bubble chart showing the number of positive cells (y‐axis) for M1 phenotypic markers (CD80, CD86). The radius of the bubbles is proportional to the MFI. b) Confocal images showing CD80 (magenta), CD86 (red) and nuclei (DAPI) inside the synovial micro‐tissues on Day_syn_ 3 and Day_syn_ 7. Scale bar 100µm. c) Multiplexed ELISA (Luminex) of factors released by synovium only cultures from Daysyn 0 to Daysyn 3 and from Daysyn 4 to Daysyn 7, comparing control samples (‘Untreated’) and samples treated with pro‐inflammatory stimulation (“Inf”). n≥3. ^*^ *p*<0.05, ^**^ *p*<0.01.

Flow cytometry analysis was performed to further evaluate the expression of inflammatory markers CD80 and CD86 at the protein level (Figure S5). Specifically, live cells were identified using physical parameters, and CD45 expression was used to isolate macrophages from the mixed population (i.e., to exclude SFBs), allowing selective evaluation of CD45+ cells. At Day_syn_ 0, the M1 markers CD80 and CD86 were expressed in 27.11% and 2.24% of macrophages, respectively. The percentage of CD80^+^ cells increased after three days of culture both in untreated (91.30%) and inflamed (95.79%) samples, suggesting that 3D culture in the JoC platform affects the expression of this marker. Although most CD45^+^ cells were CD80^+^ in both conditions at Day_syn_ 3, a significantly higher MFI was detected in inflamed samples, indicating elevated expression levels. This trend was maintained also at Day_syn_ 7. Differently, CD86 expression remained very low in untreated samples both at Day_syn_ 0 and at Day_syn_ 3. Pro‐inflammatory stimulation induced a significant increase in both the percentage of CD86^+^ cells and MFI at Day_syn_ 3 compared to controls. Unlike CD80, this trend was not maintained at Day_syn_ 7. For clarity, these data are summarized in a bubble chart (**Figure** **5**a), showing the proportion of marker‐positive cells (y‐axis) and mean fluorescence intensity (bubble size) at Day_syn_ 3 and Day_syn_ 7.

The expression of CD80 and CD86 was further assessed through immunofluorescence staining, to confirm that enzymatic digestion for flow cytometry did not compromise protein detection. As shown in Figure 5b, results were in agreement with flow cytometry, with the exception of CD80 signal in unstimulated cells, which was detected by flow cytometry but not by immunofluorescence, most likely due to intrinsic methodological differences and lower sensitivity of the latter. Nevertheless, the immunofluorescence results confirmed that macrophages responded to the pro‐inflammatory stimulation by upregulating CD80 and CD86. At Day_syn_ 3, immunofluorescence revealed a notably higher presence of CD80 and CD86 in the inflamed synovial micro‐tissues, compared to untreated controls. This difference was maintained for CD80 at Day_syn_ 7, while CD86 returned to baseline.

To complement gene and protein‐level assessments of inflammatory and phenotypic markers, a Luminex assay was performed to quantify the secretion of key soluble factors from synovial micro‐constructs under untreated and inflamed conditions, both from Day_syn_ 0 to Day_syn_ 3, and from Day_syn_ 4 to Day_syn_ 7 (Figure 5c). Notably, IL6 and IL8 displayed an increasing trend in inflamed samples at both timepoints, partially reflecting the gene expression data. MMP1 showed a significant increase from Day_syn_ 0 to Day_syn_ 3, which was not maintained from Day_syn_ 4 to Day_syn_ 7, where only a non‐significant upward trend persisted. Moreover, the chemokines CCL2 and CCL5, known to be upregulated in the synovial fluid of OA patients and involved in monocyte recruitment and macrophage activation,^[^ ^31^, ^48^, ^49^ ^]^ were significantly elevated from Day_syn_ 0 to Day_syn_ 3 in inflamed samples, while from Day_syn_ 4 to Day_syn_ 7 they remained slightly increased but without statistical significance. CCL3 was conversely not regulated in either time points. Soluble ICAM‐1 levels were significantly elevated in inflamed samples from Day_syn_ 0 to Day_syn_ 3, with a partial maintenance of this increase from Day_syn_ 4 to Day_syn_ 7, supporting the presence of a sustained inflammatory profile.^[^ ^50^ ^]^ Conversely, IL10 remained low in all conditions, consistent with the limited activation of anti‐inflammatory programs.^[^ ^51^ ^]^

In conclusion, these results suggest that the treatment with TNFα and IFNγ induced inflammatory traits and enhanced macrophage polarization toward pro‐inflammatory phenotype M1, recapitulating a key feature of OA synovitis.^[^ ^52^ ^]^ To some extent, this phenotypic shift remained stable after the removal of the stimulating agents.

### 2.6 Co‐Culture of Cartilage and Synovial Micro‐Constructs in the JoC Platform

**Figure 6:**
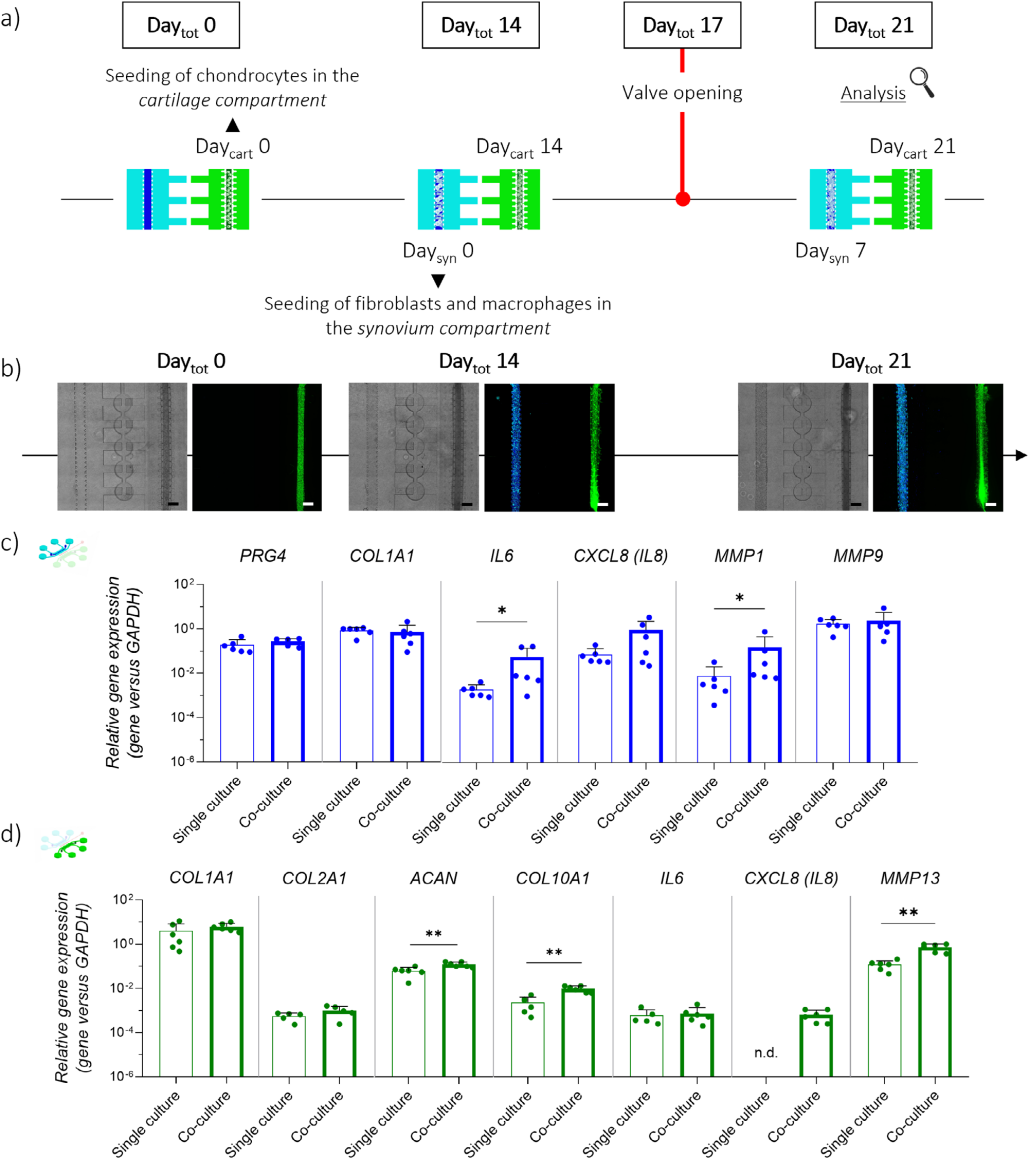
Co‐culture of cartilage and synovial micro‐constructs in the JoC platform. a) Experimental plan. hACs were injected in the cartilage compartment on Day_tot_ 0 (i.e., Day_cart_ 0) and cultured up to Day_tot_ 21 (i.e., Day_cart_ 21). On Day_tot_ 14 (i.e., Day_cart_ 14, Day_syn_ 0), MΦs and SFBs embedded were injected in the synovium compartment and co‐cultured in the platform up to Day_tot_ 21 (i.e., Day_cart_ 21, Day_syn_ 7). On Day_tot_ 17, communication valves were open. b) Fluorescence and brightfield images showing the three cell populations in the JoC platform over the culture period. Cells were stained with Vybrant™ DyeCycle™. Colors were adapted for imaging purpose (hACs in green, MΦs in blue, SFBs in light blue). Scale bar 500 µm. c) Gene expression analysis of synovial micro‐constructs on Day_tot_ 21, comparing single culture with mix of medium supplied from Day_tot_ 17, and co‐culture. d) Gene expression analysis of cartilage micro‐constructs on Day_tot_ 21, comparing single culture with mix of medium supplied from Day_tot_ 17 and co‐culture. Relative gene expression is shown, i.e., expression of gene of interest normalized against the expression of reference gene (GAPDH). Data are plotted as mean + SD (n = 6). *p<0.05, **p<0.01.

After establishing protocols to generate mature 3D human cartilage and synovial micro‐constructs and to independently induce inflammatory traits in each tissue, the JoC platform was exploited to assess the inter‐tissues crosstalk under both physiological and pathological conditions. The experimental plan and timeline for the co‐culture of cartilage and synovium micro‐tissues were first optimized, exploiting compartmentalization to comply with specific maturation timings and culture media. Briefly, hACs were seeded in the cartilage compartment on Day_tot_ 0 and allowed to mature for 14 days as previously described, while MΦs and SFBs were seeded in the synovial compartment on Day_tot_ 14, maintaining *communication valves* closed. *Communication valves* were opened on Day_tot_ 17 to enable diffusion between the micro‐constructs, and the co‐culture continued until Day_tot_ 21, to evaluate the potential impact of a direct fluidic communication between the micro‐constructs in physiological conditions (**Figure** **6**a,b). Gene expression analysis on synovial micro‐tissues revealed that the sole presence of cartilage constructs did not affect *PRG4, COL1A1* and *MMP9* expression in synovial tissues (Figure 6c, *“Co‐culture”* vs *“Single culture”*). However, a significant upregulation of *IL6* and *MMP1*, as well as an increasing trend for *IL8*, were detected in synovium samples communicating with cartilage tissues, compared to synovium‐only controls (“*Co‐culture*” vs “*Single culture*”). Then, gene expression analysis performed on cartilage tissues (Figure 6d) showed that the presence of a healthy synovium tissue did not induce changes in the expression levels of *COL1A1, COL2A1* and *IL6* when compared to cartilage‐only controls (“*Co‐culture*” vs “*Single culture*”). However, an increasing trend was noticed for *IL8* in cartilage micro‐tissues interacting with synovial micro‐tissues, compared to cartilage‐only cultures. Moreover, a significant upregulation of *ACAN, COL10A1* and *MMP13* was detected. Overall, these results revealed distinct gene expression patterns in both synovium and cartilage tissues when co‐cultured, emphasizing the potential influence of their interaction on certain key markers associated with tissue health and inflammation.

### 2.7 Effects of Synovial Inflammation on Healthy Cartilage Micro‐Tissues

**Figure 7:**
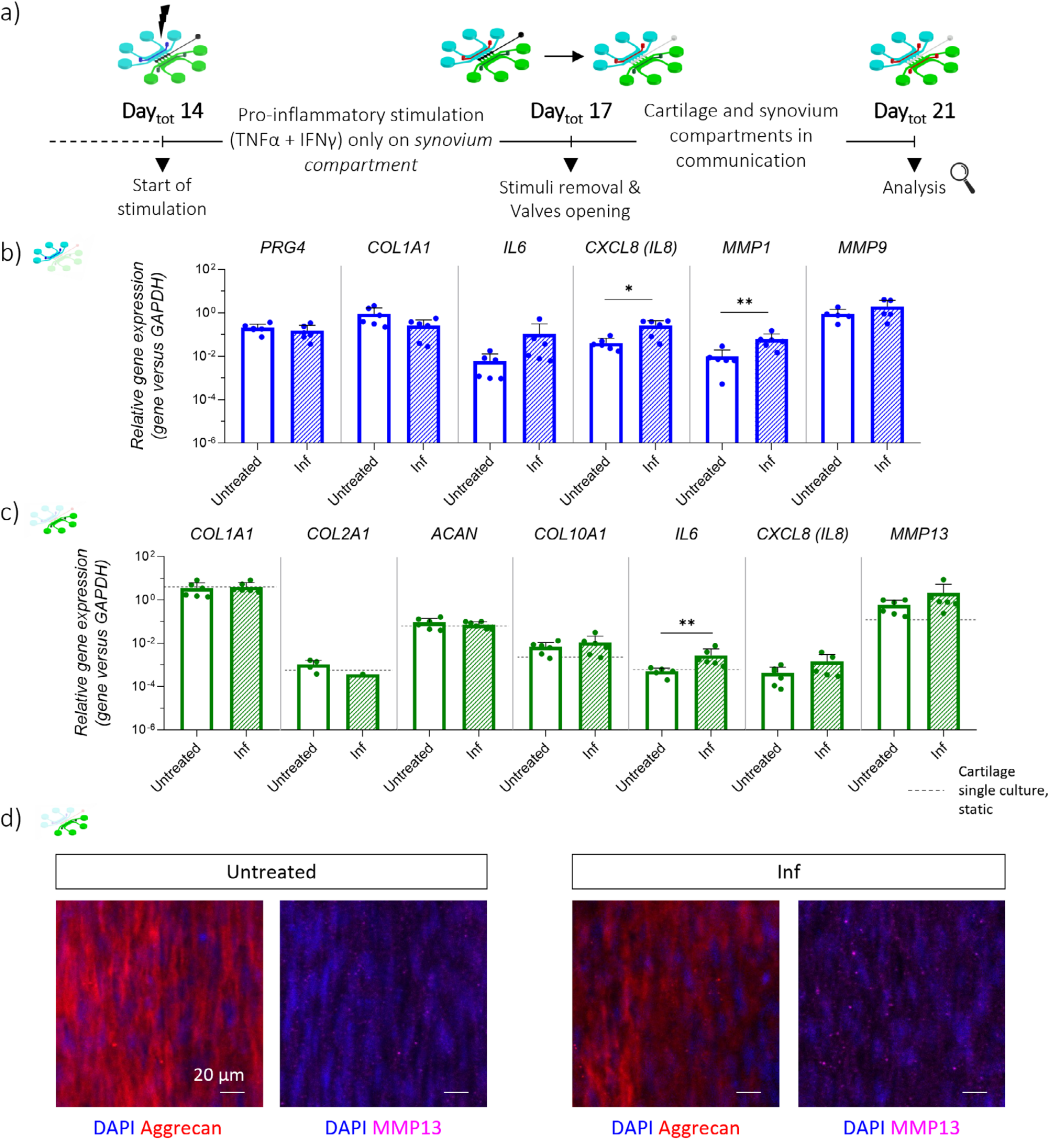
Effects of synovial inflammation on healthy cartilage micro‐tissues. a) Experimental plan. hACs were seeded in the cartilage compartment, while MΦs and SFBs in the synovium compartment as previously optimized. From Day_tot_ 14 to Day_tot_ 17, a pro‐inflammatory stimulation (100 ng mL^−1^ IFNγ, 100 ng mL^−1^ TNFα) was supplied to the synovium compartment. On Day_tot_ 17, pro‐inflammatory stimulation was removed, and communication valves were open. Analyses were conducted on synovium and cartilage micro‐tissues on Day_tot_ 21. b) Gene expression analysis of synovial micro‐constructs on Day_tot_ 21, comparing non‐stimulated synovium micro‐tissues in communication with cartilage tissues (“Untreated”), and inflamed synovium micro‐tissues in communication with cartilage tissues (“Inf”). c) Gene expression analysis of cartilage micro‐constructs on Day_tot_ 21, comparing cartilage micro‐tissues in communication with non‐stimulated synovium micro‐tissues (“Untreated”), and with inflamed synovium micro‐tissues (“Inf”). Relative gene expression is shown, i.e., expression of gene of interest normalized against the expression of reference gene (GAPDH). Data are plotted as mean + SD (n = 6, undetected values are not represented). ^*^ *p*<0.05, ^**^ *p*<0.01. Dashed lines refer to cartilage monoculture under static conditions, with mix of media supplied from Day_tot_ 17 (i.e., “Single culture” of Figure 6d). d) Confocal images of cartilage micro‐tissues, showing nuclei (DAPI, blue), aggrecan (red), and MMP13 (magenta), comparing micro‐tissues in communication with non‐stimulated synovium micro‐tissues (“Untreated”) and micro‐tissues in communication with inflamed synovium micro‐tissues (“Inf”). Scale bar 20 µm.

After optimizing the co‐culture protocol for synovium and cartilage tissues, the established JoC platform was exploited to unravel the effects of inflamed synovial micro‐constructs on healthy cartilage micro‐tissues. Based on the established protocol, hACs were seeded in the *cartilage compartment* on Day_tot_ 0, and MΦs and SFBs were seeded in the *synovium compartment* on Day_tot_ 14, with *communication valves* kept closed. On Day_tot_ 14, inflammatory stimulation (i.e., TNFα and IFNγ) was applied to synovial constructs until Day_tot_ 17, when it was removed and replaced with fresh culture medium. On the same day, *communication valves* were opened to initiate co‐culture, which continued until Day_tot_ 21 (**Figure** **7**a). The initial phase involved conducting gene expression analysis on both untreated and inflamed synovial microtissues to ascertain the persistence of the pro‐inflammatory phenotype in the *synovial compartment* upon inflammatory stimulation in the complete system (Figure 7b). No changes in *PRG4* expression were detected in inflamed synovium samples compared to healthy synovium samples (“*Inf*” vs “*Untreated*”). In contrast, inflammatory stimulation led to slight *COL1A1* and *PRG4* downregulation, significant upregulation of *IL8* and *MMP1*, and an increasing trend for *IL6* and *MMP9*.

Subsequently, gene expression and immunofluorescence analyses were performed on cartilage‐microtissues recovered on Day_tot_ 21 to investigate whether inflammation in the synovium triggers degenerative responses in cartilage. As shown in Figure 7c, no variations were observed in the expression of *COL1A1, ACAN*, and *COL10A1* between cartilage samples interacting with inflamed synovial tissues and with healthy synovial tissues (“*Inf*” vs “*Untreated*”). Conversely, cartilage samples in communication with inflamed synovial tissues exhibited a reduced expression of *COL2A1*, as well as a significantly higher expression of *IL6* and an increasing trend for *IL8* and *MMP13*. This was corroborated by Luminex‐based quantification of cartilage‐secreted factors, which revealed an increased release of pro‐inflammatory cytokines (IL6, IL8), along with a reduction in the anti‐inflammatory cytokine IL10 and in TIMP1, a natural inhibitor of matrix metalloproteinases,^[^ ^53^ ^]^ when exposed to inflamed synovium (Figure S6a). Finally, at the protein level, *“Untreated”* samples exhibited a matrix rich in aggrecan, along with intracellular expression of MMP13 (Figure 7d). Synovial inflammation resulted in a modest reduction in aggrecan deposition and a slight raise in MMP13 expression. Overall, these findings indicate that synovial inflammation alters the expression of critical cartilage markers and may contribute to inflammatory processes within cartilage tissue.

### 2.8 Effects of OA HPC Cartilage on Healthy Synovium Micro‐Tissues

**Figure 8:**
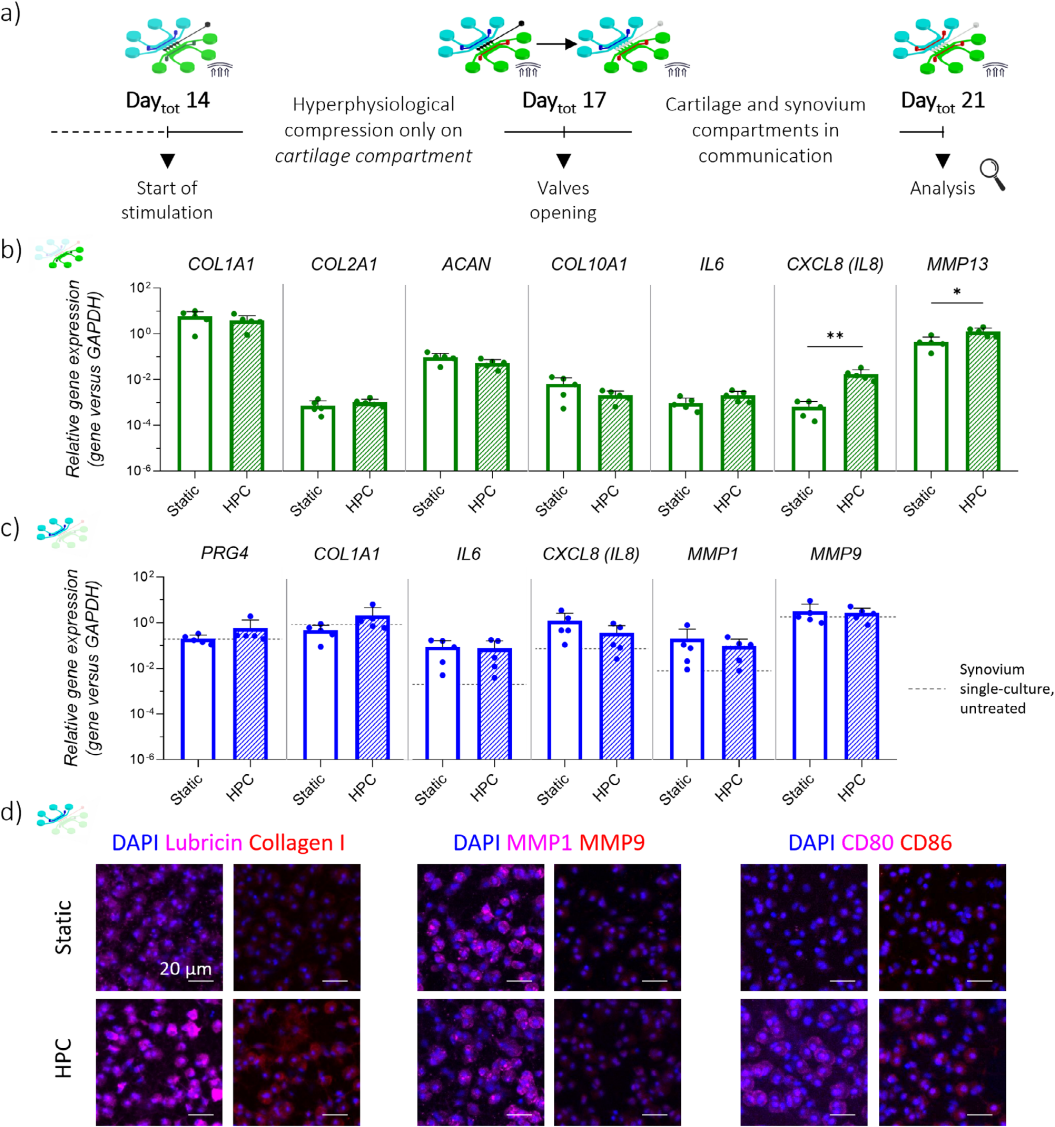
Effects of OA HPC cartilage on healthy synovium micro‐tissues. a) Experimental plan. hACs were seeded in the cartilage compartment, while MΦs and SFBs in the synovium compartment as previously optimized. From Day_tot_ 14, a HPC was applied to the cartilage compartment. On Day_tot_ 17, communication valves were open. Analyses were conducted on synovium and cartilage micro‐tissues on Day_tot_ 21. b) Gene expression analysis of cartilage micro‐constructs on Day_tot_ 21, comparing static cartilage micro‐constructs in communication with synovium tissues (“Static”) and HPC cartilage micro‐tissues in communication with synovium tissues (“HPC”). c) Gene expression analysis of synovium micro‐constructs on Day_tot_ 21, comparing synovium micro‐tissues in communication with static cartilage micro‐constructs (“Static”), and with HPC cartilage micro‐tissues (“HPC”). Relative gene expression is shown, i.e., expression of gene of interest normalized against the expression of reference gene (GAPDH). Data are plotted as mean + SD (n = 5). *p<0.05, **p<0.01. Dahsed lines refer to synovium only cultures monoculture under untreated conditions, with mix of media supplied from Day_tot_ 17 (i.e., “Single culture” of Figure 6c). d) Confocal images of synovium micro‐tissues, showing lubricin and collagen type‐I on the left, MMPs in the center, and CD80 and CD86 on the right, comparing micro‐tissues in communication with static cartilage micro‐tissues (“Static”) and micro‐tissues in communication with HPC cartilage micro‐tissues (“HPC”). Scale bar 20 µm.

As a second step, the JoC platform was applied to investigatel the effects of HPC‐induced OA‐like cartilage micro‐constructs on healthy synovium micro‐tissues (**Figure** **8**a). In details, hACs, and MΦs/SFBs were seeded in their respective culture compartments as described above, with *communication valves* kept closed. A cyclic HPC was applied exclusively to the *cartilage compartment*, from Day_tot_ 14 to Day_tot_ 21. Communication valves were opened from Day_tot_ 17 to Day_tot_ 21.

The direct effect of HPC on cartilage micro‐tissues co‐cultured with synovium was first assessed (Figure 8b). HPC cartilage samples in communication with synovium tissues (“*HPC*”) showed reduced expression of *COL10A1* and *ACAN* compared to static cartilage samples interacting with synovium constructs (“*Static*”). No relevant differences were detected in *COL1A1* and *COL2A1* expression. Conversely, HPC induced an increase in *IL6* expression, as well as a significant upregulation of *IL8* and *MMP13*, confirming that, in line with previous findings, HPC exerts a direct catabolic effect on cartilage microtissues, even in a co‐culture setup.

Second, gene expression analysis and immunofluorescence were performed on synovial samples to evaluate the indirect effect of HPC‐stimulated cartilage on synovial microtissues following valve opening. As depicted in Figure 8c, synovial tissues in contact with HPC cartilage constructs (“*HPC*”) showed an increasing trend in the expression of *PRG4* and *COL1A1*, in comparison with synovial tissues exposed to static cartilage (“*Static*”). Conversely, *IL8* and *MMP1* were slightly downregulated in “*HPC*” samples, while no differences were observed for *IL6* and *MMP9* expression. These results were confirmed at protein level, through immunofluorescence staining. A higher presence of lubricin and collagen type‐I was appreciated in “*HPC*” samples compared to “*Static*” samples, while no or slight differences could be observed in the intracellular signals of *MMP1* and *MMP9* (Figure 8d). Moreover, macrophage activation was assessed at protein level through immunofluorescence of M1 phenotypic markers: “*HPC*” samples were characterized by a higher occurrence of the pro‐inflammatory markers CD80 and CD86. Finally, quantification of synovium‐secreted factors (Figure S6b) demonstrated a reduction in IL6, IL8 following co‐culture with HPC cartilage, alongside a decrease in TIMP1, thus confirming gene expression. Notably, osteopontin, a glycoprotein involved in tissue remodeling and early fibrotic responses, showed a moderate increase.^[^ ^54^ ^]^ Overall, these findings suggest that mechanical stress applied to cartilage initiates a secondary response in synovial tissues, marked by macrophage activation and early indicators of tissue remodeling.

### 2.9 Summary of Experimental Findings

To facilitate the comparison among healthy, mechanically stressed, and inflamed conditions across the different experimental setups, both in single and co‐culture, a comprehensive summary of the observed molecular and phenotypic changes is provided in Table S1.

## 3 Discussion

The primary barrier in the development of pharmacological interventions capable of reversing OA progression is the limited understanding of the pathogenic mechanisms initiating the disease and of the interactions among various joint tissues.^[^ ^55^ ^]^ In this scenario, the concept of a joint‐on‐chip model represents a significant advancement in OA research, as animal models do not accurately replicate human OA pathology and traditional in vitro models fail to reproduce the complexity of joint environments.^[^ ^56^ ^]^ The importance of taking into account reciprocal tissue cross‐talk in joint models was previously underlined by Rothbauer et al., that developed a chip‐based chondro‐synovial organoid model using primary human chondrocytes and SFBs from patients with rheumatoid arthritis.^[^ ^57^ ^]^ However, the study primarily examined a pathology distinct from OA and provided limited insights into the effects of pathological SFBs on chondral organoids. Another example emphasizing the significance of tissue cross‐talk is represented by the mesenchymal stem‐cell derived miniJoint, in which a synovitis model was first recapitulated by exposing the synovial fibrous tissue to IL1β, followed by assessment of its impact on osteochondral and adipose tissues.^[^ ^35^ ^]^ Interestingly, the osteochondral complex showed signs of OA induction, such as downregulation of collagen type‐II and aggrecan, and upregulation of IL8 and MMP13. However, while the miniJoint platform demonstrated considerable promise for OA investigation, it lacked the incorporation of mechanical stimulation, which is a critical factor in joint function and pathology, and of an immune component, which limits its relevance in studying immune‐mediated aspects of OA. Moreover, communication between tissue modules was obtained through a shared medium flowing unidirectionally from the synovial tissue to the osteochondral complex; therefore, the miniJoint platform did not consider the bidirectional cross‐talk naturally occurring in vivo. Another relevant contribution is the personalized joint‐on‐a‐chip model recently proposed by Petta et al.^[^ ^34^ ^]^, which included hydrogel‐based cartilage and synovium compartments, perfused through a central synovial fluid channel. The authors reproduced key OA traits by exposing patient‐derived tissues to OA synovial fluid, leading to increased production of inflammatory cytokines such as IL8 and TNFα, as well as upregulation of degradative enzymes including MMP1 and MMP13 in both SFBs and chondrocytes. Additionally, the model enabled the screening of orthobiologics. While the platform is promising for personalized therapy testing, certain limitations restrict its utility for mechanistic investigations of OA pathogenesis, namely, the lack of mechanical stimulation and immune components, as well as the inability to dissect causal interactions between tissues.

Building on these considerations, we developed a novel JoC platform, to generate a highly controlled, physiologically relevant model for studying the interaction between mechanically active cartilage and immune‐competent synovium during OA onset. Unlike earlier platforms published by our groups,^[^ ^29^, ^38^ ^]^ which were either limited to single‐tissue cultures or unable to capture the complex dynamic cross‐talk among joint compartments, this new system enables co‐culture and controlled, bidirectional communication between cartilage and synovium. Leveraging valve‐based compartmentalization, this technological innovation allows for an unprecedented dissection of inter‐tissue crosstalk under physiologically relevant conditions. Specifically, the JoC was designed to keep the compartments separated during their individual maturation, by using normally closed valves, thus accommodating different maturation timelines and media compositions. Moreover, the proposed design supports independent mechanical and biochemical stimulation of *cartilage* and *synovium compartment*, respectively. This is crucial because OA is influenced by both alteration in mechanical forces (e.g., 30% hyperphysiological HPC compression^[^ ^29^ ^]^) and biochemical changes (e.g., inflammation, enzymatic degradation^[^ ^58^ ^]^). Being able to simulate these conditions independently is key to dissecting their individual contributions to OA progression. Finally, the inclusion of a paracrine communication system between the two compartments, finely controlled by valves’ opening at defined time points, mimics the in vivo environment where cartilage and synovium tissues interact through signaling molecules via synovial fluid.^[^ ^59^ ^]^

Following the design and technical validation of the JoC platform, biological qualification was performed to investigate whether mechanical damage to the cartilage triggers inflammatory responses in a healthy synovium, and conversely, whether an inflamed synovium initiates cartilage degradation. To this end, we initially fine‐tuned individual models for cartilage and synovium, along with the induction of OA traits in each compartment. Subsequently, we evaluated the impact of co‐culturing healthy cartilage and synovium micro‐tissues, and finally, evaluated their interplay under pathological conditions.

As for the cartilage‐on‐chip model, we first validated the replication of a mechanically induced OA phenotype previously established by our group^[^ ^29^ ^]^ within the newly proposed JoC platform. In particular, cartilage micro‐constructs were successfully generated using human primary hACs embedded in fibrin gel, as demonstrated by the upregulation of *PRG4*, *ACAN* and *COL2A1* at the end of the 14‐day maturation period, as well by the deposition of collagen type II and aggrecan observed through immunofluorescence analysis. Our findings were in line with data from other cartilage‐on‐chip studies.^[^ ^23^, ^26^, ^27^, ^29^ ^]^ Subsequently, the application of HPC to mimic daily walking stresses and induce OA traits brought to findings that aligned with the study of Occhetta et al.^[^ ^29^ ^]^ Remarkably, the significant upregulation of pro‐inflammatory gene *IL6* and *MMP13* in HPC samples with respect to static controls, along with trends in *IL8* expression, are in line with the inflammatory and degradative processes observed in OA joints.^[^ ^20^ ^]^ These results also align with the study of Liu et al.^[^ ^28^ ^]^, where cyclic magneto‐mechanical stimulation induced MMP13 upregulation in a cartilage‐on‐chip model. Interestingly, similar increases in *IL8* and *MMP13* expression were also reported by Petta et al. when exposing cartilage constructs to osteoarthritic synovial fluid.^[^ ^34^ ^]^ Notably, gene expression levels of *ACAN* and *COL2A1* remained stable, suggesting that catabolic activity was not compensated by new matrix synthesis. This was confirmed at the protein level, where the decreased expression of aggrecan in HPC samples compared to controls confirmed the onset of degradative changes and cartilage breakdown typical of OA.^[^ ^60^ ^]^ Finally, the reduction in SOX9 concentration, i.e., a transcription factor that is essential for cartilage ECM formation and whose disfunction is often detected in OA,^[^ ^61^, ^62^ ^]^ further underscores the shift toward an OA‐like state in the mechanically stressed constructs, consistent with previous observations in IL1β‐induced OA models.^[^ ^27^ ^]^

Then, a 3D model of synovium was successfully established within the *synovial compartment* of the JoC platform. As compared to other models,^[^ ^34^, ^35^, ^63^ ^]^ where synovial constructs were recapitulated by using SFBs only, here SFBs were co‐cultured together with MΦs in an animal‐serum free medium to better resemble the multicellular in vivo environment found in the synovial membrane.^[^ ^64^ ^]^ Moreover, compared to the study of Mondadori et al., where SFBs were cultured in a microfluidic platform for less than 48 h,^[^ ^38^ ^]^ here, the culture period was extended to one week to promote synovial tissue maturation, while preserving macrophage viability and functionality, also in line with previous studies employing 7‐day culture periods in macrophage‐containing 3D systems.^[^ ^65^, ^66^, ^67^ ^]^ Indeed, monocyte‐derived primary human macrophages are terminally differentiated, non‐proliferative cells,^[^ ^68^ ^]^ and are known to undergo senescence‐related changes and functional decline when maintained in prolonged cultures.^[^ ^69^ ^]^ High cell viability throughout the culture period suggests that the 3D microenvironment within the JoC platform supports cell health. Moreover, the gene expression dynamics highlights the model ability to replicate the biological processes of synovial tissue. Specifically, the stable expression of PTPRC (CD45) throughout the culture period indicates the maintenance of a consistent ratio of MΦs to SFBs, which is key to ensure the integrity of the model. Moreover, increased expression of genes associated with synovial ECM components (*COL1A1, COL4A1*) and of *PRG4*, which encodes lubricin, a protein essential for joint lubrication,^[^ ^70^, ^71^, ^72^ ^]^ indicated appropriate tissue maturation, aligning with findings by Li et al. and Petta et al.^[^ ^34^, ^35^ ^]^ The active ECM production was confirmed by the increased deposition of collagen type‐I at protein level.

Subsequently, we aimed at recapitulating an inflammatory state in the synovial micro‐constructs, which is usually found in both early and late stages in most of OA patients.^[^ ^73^, ^74^ ^]^ Since M1 macrophages, also known as “classically activated macrophages”, are key contributors to the secretion of molecules involved in joint inflammation,^[^ ^74^ ^]^ we adapted an established protocol to induce macrophage polarization toward the M1 phenotype using 100 ng mL^−1^ TNFα and 100 ng mL^−1^ IFNγ.^[^ ^45^ ^]^ IFN‐γ is a key Th1‐derived cytokine for M1 polarization, and its combination with TNF‐α leads to a synergistic enhancement of pro‐inflammatory macrophage activation through NF‐κB and STAT1 signaling pathways.^[^ ^75^, ^76^ ^]^ Of note, this cytokine combination has been previously used to treat synovial explants, demonstrating the critical role of macrophages in synovial inflammation and providing a valuable framework for testing anti‐inflammatory drugs.^[^ ^77^ ^]^ Moreover, conditioned medium from macrophages stimulated with TNFα and IFNγ has been shown to induce a strong inflammatory and catabolic response in cartilage explants, supporting the hypothesis that M1 macrophages directly contribute to cartilage damage.^[^ ^78^ ^]^ In line with previous observations, inflammation with TNFα and 100 ng mL^−1^ IFNγ reliably induced M1 polarization in our model, as confirmed by CD80 upregulation at gene and protein levels. Interestingly, at the protein level, a substantial increase in CD80 expression was observed in untreated samples after three days of culture within the platform, suggesting that the 3D culture synovial microenvironment itself might already contribute to a certain degree of macrophage activation in the absence of inflammatory stimuli. Fibroblast‐ and matrix‐derived factors have indeed been shown to modulate macrophage phenotype in previous studies, supporting this hypothesis. For instance, conditioned medium from cancer‐associated fibroblasts has been shown to selectively increase CD80 expression in M0 macrophages, without affecting CD86 levels.^[^ ^79^ ^]^ Moreover, fibrin‐derived components, such as fibrinogen, might also contribute to macrophage activation through TLR4 signaling,^[^ ^80^ ^]^ a pathway that directly induces CD80 expression.^[^ ^81^, ^82^ ^]^ In addition to these microenvironmental factors, it must also be noted that pooling monocytes from different donors may introduce a degree of biological noise, potentially contributing to such basal inflammatory activation observed in control samples. Nevertheless, this strategy has commonly been adopted within several in vitro models,^[^ ^45^, ^77^, ^78^, ^83^, ^84^ ^]^ and a recently published study suggested that monocyte‐derived macrophages pooled from different donors retain functional stability, while helping to reduce inter‐donor variability.^[^ ^85^ ^]^ Nonetheless, the presence of TNFα and IFNγ significantly enhanced macrophage activation in our model, as evidenced at the protein level for both pro‐inflammatory markers CD80 and CD86. Of note, immunofluorescence stainings were in accordance with flow cytometry analysis: thus, for sake of simplicity, immunofluorescence was chosen for subsequent analyses of macrophage phenotypic markers. Moreover, the significant upregulation of pro‐inflammatory and pro‐degradative genes such as *IL6*, *IL8* and *MMP1* in the inflamed samples at Day_syn_ 3, with a partial maintenance of this trend at Day_syn_ 7, is consistent with the inflammatory profile of synovitis and with the findings obtained by Thompson et al. in the synovium‐on‐a‐chip, where co‐cultured synoviocytes and endothelial cells under mechanical loading were stimulated with IL1β.^[^ ^86^, ^87^ ^]^ Conversely, the downregulation of *PRG4* in inflamed samples is in discordance with other studies reporting that SFBs secrete more lubricant molecules when treated in vitro with IL1β and TNFα.^[^ ^88^ ^]^ Furthermore, the decrease in *COL1A1* expression reflects ECM degradation, but contrasts with reports of synovial fibrosis associated to synovitis, which typically involves collagen type I accumulation.^[^ ^59^ ^]^ Overall, these findings indicate that the pro‐inflammatory stimulation on synovial micro‐constructs induced an increased expression of inflammatory cytokines and a decreased expression of structural tissue components. This aligns with the inflammatory profile of synovitis, but not with synovial fibrosis, suggesting that the fibrotic cascade could not initiate due to the chosen treatment or to the relatively short duration of the experiment. Importantly, while most pro‐inflammatory, pro‐degradative, and structural genes tended to return to baseline levels after stimulus withdrawal, the expression of macrophage markers remained more stable over time, suggesting a plastic fibroblast response and a more persistent inflammatory macrophage phenotype.

The gene inflammatory profile was further substantiated at the protein level by quantification of secreted cytokines and chemokines. Increasing trends of IL6 and IL8 were in line with gene expression, and the upregulation of MMP1 secretion confirmed the acquisition of a degradative phenotype. In contrast, the anti‐inflammatory cytokine IL10 remained low throughout, supporting polarization toward a pro‐inflammatory M1‐like phenotype.^[^ ^51^ ^]^ Additionally, the significant increased secretion of CCL2 and CCL5 in inflamed samples corroborates previous findings reporting high levels of these chemokines in the synovial fluid of OA patients and their role in monocyte recruitment and macrophage activation.^[^ ^48^, ^49^ ^]^ Similar trends were also observed in a microfluidic vascularized osteochondral model of OA inflammation recently presented by Salehi et al.^[^ ^31^ ^]^. Interestingly, CCL2 and CCL5 levels remained mildly elevated from Day_syn_ 4 to Day_syn_ 7, suggesting a sustained chemotactic potential beyond the removal of the inflammatory stimuli. Finally, ICAM‐1 followed a similar temporal pattern, with significant upregulation at early timepoints and partial maintenance thereafter. Although the functional role of soluble ICAM‐1 is less clearly defined, its increased levels are broadly associated with chronic inflammation and have been proposed as surrogate markers in other inflammatory disorders.^[^ ^50^ ^]^ Overall, this analysis provided an additional layer of validation for the inflammatory state induced in our synovium model, with several pro‐inflammatory features partially persisting even after stimulus withdrawal. This is crucial to enable the assessment of synovium‐driven effects on cartilage during co‐culture, without the confounding influence of exogenously supplied cytokines.

After independently characterizing the two tissues, the JoC platform was exploited to co‐culture cartilage and synovial micro‐tissues, underscoring the importance of compartmentalization in maintaining distinct environments to address the biological requirements of these tissues. The 14‐day maturation period for hACs within the *cartilage compartment* indeed reflects the need for prolonged culture to achieve mature cartilage tissues in vitro.^[^ ^89^ ^]^ In contrast, as discussed above, MΦs do not tolerate such prolonged culture periods; thus the possibility to seed MΦs and SFBs in the *synovial compartment* two weeks after chondrocytes –owing to compartmentalization– is crucial for complying with those specific different maturation timings.

Initially, the evaluation focused on elucidating the interplay between healthy cartilage and synovium constructs. As revealed by gene expression analysis, healthy cartilage constructs elicited an increase in pro‐inflammatory and pro‐degradative genes in synovial micro‐tissues, suggesting a level of activation even in physiological conditions, in contrast to what obtained from Li et al.^[^ ^35^ ^]^ This observation suggests that even in the absence of external stimulation, cartilage constructs may release soluble factors capable of eliciting a mild activation in synovial micro‐tissues. Further studies will be required to dissect the specific contribution of such cartilage‐derived cues. Moreover, the interaction of cartilage with healthy synovial micro‐tissue led to a slight increase in collagen type‐II and a significant increase in aggrecan expression, suggesting a supportive role of the synovium in maintaining cartilage health, as also stated by Rothbauer et al.^[^ ^57^ ^]^ However, an increase in the expression of *COL10A1*, *IL8* and *MMP13* was also shown, pointing toward possible early signs of hypertrophic‐like changes in cartilage upon interaction with synovial tissue, even in the absence of inflammation. Future studies extending the co‐culture duration and targeting additional markers of hypertrophy will be crucial to better elucidate this phenomenon.

Once fully validated, the JoC platform was used to investigate cartilage‐synovium crosstalk during OA. As previously mentioned, OA is a low‐grade chronic inflammatory disease, where synovitis is a hallmark of both early and late stages, contributing to OA development and progression.^[^ ^90^, ^91^ ^]^ However, underlying mechanisms of synovitis remain poorly understood.^[^ ^92^ ^]^ In this study, we aimed to determine whether synovitis alone independently drives signs of cartilage inflammation and degradation. First, gene expression analysis on synovial micro‐constructs suggested that the interaction with cartilage micro‐constructs had an exacerbating effect on synovial inflammation, as the differences evidenced between inflamed and untreated samples in complete chips were more pronounced compared to those observed in the characterization of inflammation within synovium‐only cultures. This finding suggests a potential role of cartilage in the perpetuation of synovial inflammation. On the other hand, analyses performed on cartilage micro‐tissues revealed a higher expression of *IL6, IL8* and *MMP13* in cartilage co‐cultured with inflamed synovium, suggesting early signs of cartilage catabolism and inflammation, in accordance with the results obtained in the miniJoint.^[^ ^35^ ^]^ Notably, the transcriptional upregulation of IL6 and IL8 was confirmed at the protein level by Luminex‐based cytokine quantification. These results were accompanied by a decreased expression of *COL2A1*, while no relevant findings were revealed for *COL1A1, ACAN* and *COL10A1*. Interestingly, immunofluorescence staining indicated aggrecan content decrease in cartilage tissues in contact with inflamed synovium, and a slight increase in MMP13 expression. In addition, Luminex profiling highlighted a reduction in the anti‐inflammatory cytokine IL10 and in TIMP1, reflecting the disrupted MMP/TIMP balance characteristic of OA, where inadequate TIMP1 fails to regulate matrix degradation.^[^ ^93^ ^]^

Overall, transcriptional, secretory, and protein‐level data indicate that synovial inflammation contributes to the induction of inflammatory traits in healthy cartilage, initiating early signs of matrix remodeling. These results provide interesting preliminary insights on the impact of synovitis on initiating inflammatory processes in cartilage tissue, but further investigation is needed to demonstrate that synovitis has an important role in the structural degradation of cartilage during OA.^[^ ^91^ ^]^ It is important to underline that the findings presented here are strictly dependent on the specific inflammatory conditions applied in our model. Although TNF‐α shares mechanisms of action with other pro‐inflammatory cytokines, activating NF‐κB and MAPK pathways, and promoting the expression of catabolic enzymes and inflammatory mediators, our observations cannot be generalized to other inflammatory settings without further validation. In this context, future work should aim to test the effects of additional pro‐inflammatory cytokines such as IL1β, widely applied in OA in vitro models^[^ ^31^, ^35^, ^94^ ^]^ or even explore more complex stimuli such as pathological synovial fluid derived from OA patients.^[^ ^34^ ^]^ These extensions would be particularly valuable to assess whether different inflammatory milieus might differentially affect macrophage and synovial fibroblast behavior and their crosstalk with cartilage tissue.

Conversely, one of the prevailing theories of OA development suggests that an initial trauma, often mechanical, triggers the release of mediators that activate various inflammatory pathways, ultimately leading to loss of cartilage integrity and joint damage.^[^ ^73^ ^]^ Here, we aimed to evaluate the impact of mechanically‐damaged cartilage on the integrity and function of healthy synovium constructs. In this context, we maintained HPC throughout the co‐culture phase to faithfully reproduce the sustained mechanical overloading commonly observed in OA patients. This differs from the previous experimental set‐up, where pro‐inflammatory stimulation in the synovial compartment was discontinued on Day_tot_ 17 to avoid a dominant inflammatory signal that could mask more subtle synovium‐derived effects on cartilage, thereby allowing a clearer assessment of tissue paracrine crosstalk. First, analysis of the *cartilage compartmen*t revealed decrease in aggrecan content and upregulation of *IL6, IL8* and *MMP13* in HPC cartilage samples interacting with synovium tissues, consistently with the observations made in the characterization of HPC within cartilage‐only cultures in this study. Interestingly, increased expression of lubricin was detected in synovial tissues in contact with HPC cartilage, confirmed both at gene and protein level, suggesting that mechanical injury to cartilage may induce an initial release of protective factors in the synovium.^[^ ^95^ ^]^ Additionally, the upregulation of collagen type‐I points toward early extracellular matrix remodeling, a phenomenon not observed in synovium‐only cultures. However, further analyses are needed to clarify whether these changes are transient or indicative of a sustained pro‐fibrotic shift, usually observed in OA patients.^[^ ^59^ ^]^ To explore this latter possibility, we measured the level of osteopontin (a glycoprotein implicated in tissue remodeling and fibrosis in multiple organs)^[^ ^54^ ^]^ in the supernatant, observing a trend toward increased secretion in synovial constructs co‐cultured with damaged cartilage. Additional studies, including extended culture durations and additional fibrotic markers, will be however necessary to elucidate this response more comprehensively.

Interestingly, co‐culture with HPC cartilage resulted in macrophage activation, as shown by increased CD80 and CD86 expression. This finding supports the notion that macrophages are key orchestrators of early inflammatory responses in OA.^[^ ^96^ ^]^ The apparent discrepancy between macrophage activation and the lack of broad cytokine and MMP induction may reflect an early‐stage immune response, where macrophage polarization toward the M1 phenotype is present but not yet sufficient to trigger a broader inflammatory cascade. Supporting this interpretation, secretome profiling revealed a reduction in IL6 and IL8. While this may suggest a limited inflammatory phenotype, the concurrent downregulation of TIMP1 could indicate an early imbalance in matrix homeostasis, where regulatory mechanisms that normally counteract matrix degradation are impaired. While we cannot definitively assign a specific clinical stage to our in vitro system, these findings are consistent with reports indicating that early OA often exhibits subtle inflammatory changes rather than robust cytokine and MMP elevations.^[^ ^7^, ^97^, ^98^ ^]^

In summary, our findings indicate that the JoC platform holds significant potential for elucidating the interactions between different joint tissues during OA development, potentially dissecting the cause‐effect relationship underlying disease onset. Our results suggest that mechanical damage of cartilage tissue may play a critical role in OA initiation, primarily through early macrophage activation. However, our findings also indicate that inflammation in the synovium alone can induce inflammatory traits in cartilage. Therefore, the current model will require optimization for extended co‐culture periods and additional analyses will be necessary, to gain a more comprehensive understanding of the long‐term interactions between cartilage and synovium, with the goal of determining whether one tissue predominates in triggering osteoarthritic changes. Additionally, to enhance the biological accuracy of the model, it would be advantageous to incorporate also resident synovial macrophages in the synovial compartment, to more faithfully recapitulate the complexity of the synovial tissue. Resident synovial macrophages represent a minority population in synovial tissue and their isolation and culture is challenged by technical issues such as low yield and fibroblast overgrowth. From a biological perspective, although resident synovial macrophages contribute to tissue homeostasis,^[^ ^99^, ^100^ ^]^ synovial inflammation is largely driven by infiltrating monocyte‐derived macrophages.^[^ ^101^ ^]^ Our choice to include only the latter thus reflects the initial intent to model the pro‐inflammatory microenvironment typical of arthritic joints. Moreover, the synovial compartment should also integrate endothelial cells, considering the frequent occurrence of angiogenesis in OA.^[^ ^102^ ^]^ More broadly, the current JoC platform does not yet recapitulate immune cell recruitment or vascular dynamics, two processes that are increasingly recognized as key contributors to joint degeneration.^[^ ^103^, ^104^, ^105^, ^106^ ^]^ This aspect has been partially addressed in the synovium‐on‐a‐chip developed by Thompson et al.^[^ ^87^ ^]^, which incorporated endothelial cells to explore monocyte recruitment under inflammatory conditions. The absence of circulating immune cells, such as monocytes or lymphocytes, limits the capacity to fully mimic the immunological complexity observed in vivo, both in the synovial membrane and in the cartilage tissue, which is known to actively participate in inflammatory signaling. Similarly, the lack of a vascular component affects not only synovial remodeling but also restricts the possibility of studying endothelial‐mediated recruitment of immune cells. These omissions may influence the extent and kinetics of inflammatory and degradative responses captured by our model. Finally, since OA leads to systemic changes in all tissues within synovial joints, including the subchondral bone would be an important step toward reproducing the full joint microenvironment. Although these additions will increase the complexity of the system, they represent a concrete future direction for expanding the JoC platform toward a more comprehensive and translationally relevant in vitro model of the joint. In particular, we have already developed vascularized osteochondral models and immune‐integration strategies using immune cells in suspension, which could be adapted to enrich future versions of the JoC platform.^[^ ^31^, ^43^ ^]^

## 4 Conclusion

Aiming to advance the understanding of OA onset, we presented the development of a compartmentalized JoC platform that enables: i) the independent maturation of 3D human cartilage and synovial micro‐structures, ii) the induction of OA characteristics in either micro‐tissue through mechanical or biochemical stimulation, and iii) precise control of their interactions over space and time. Our study highlights the critical role of tissue‐level crosstalk in OA onset and demonstrates the platform's potential as a powerful tool for translational research. By providing a dynamic and highly controlled environment, the JoC platform enables accurate replication of the complex interactions within joint environments and pathological processes, offering novel insights into OA pathogenesis. This unique platform advances our understanding of OA mechanisms and facilitates the identification of key therapeutic targets for the development of effective treatments. Furthermore, the JoC platform serves as a valuable tool for testing therapeutic strategies, allowing for the investigation of cartilage and synovium responses to various treatments and assessing whether targeting either tissue alone is sufficient to reverse disease progression.

## 5 Experimental Section

### Microfluidic Device Design

The microfluidic platform is composed of three layers of PDMS (Sylgard 184, Dow Corning): a *valve* layer, an *actuation layer* and a *culture chamber layer*. The *culture chamber layer* features two 143 µm‐high culture compartments, here named *synovium compartment* and *cartilage compartment*. The *synovium compartment* is composed of a 400 µm‐wide central channel for cell‐laden hydrogel injection and two lateral medium channels (1000 µm wide), separated by two rows of pass‐through posts with trapezoidal cross‐section (main base 160 µm, smaller base 56 µm, height 90 µm), with an inter‐post distance of 90 µm within the same row. On the other hand, the *cartilage compartment* is composed by a 300 µm‐wide central channel and two lateral medium channels (1000 µm wide), separated by two rows of T‐shaped posts, with each branch of the shape being 300 µm long and 100 µm thick. Posts height is set to 100 µm, with an underneath gap of 43 µm. Within each row, inter‐post distance is 30 µm. In both compartments, central gel channels are provided with two injection ports (1 mm diameter), while medium channels are provided with medium reservoirs (5 mm diameter) at their ends. From each of the two compartments, an array of five parallel blind channels (1000 µm x 400 µm) is designed to allow the communication between microtissues, being the five channel pairs kept separated by 250 µm PDMS walls. The *valve layer* (150 µm high) features five interconnected rounded cavities (1 mm diameter) aligned on top of the PDMS walls separating the culture compartments. Finally, the *actuation layer* is 50 µm high and features a rectangular chamber (2050 µm x 7900 µm) aligned on top of the *cartilage compartment*. To avoid collapse, the actuation chamber is provided with five rows of cylindrical pass‐through posts (radius 60 µm). Access to valves and actuation is guaranteed through 1.5 mm holes.

### Microfluidic Device Fabrication

The devices were realized through photolithography and soft lithography. First, layouts containing the desired geometric features for the different layers were realized using computer‐assisted design (CAD) software (AutoCAD, Autodesk Inc.). CAD drawings were then used to fabricate three master molds of i) *culture chamber*, ii) *valve* and iii) *actuation layers* through standard photolithography techniques in a cleanroom environment (Polifab, Politecnico di Milano). Multi‐layer photolithography was used for the fabrication of the *culture chamber layer* mold: first, a 43 µm‐high layer of SU‐8 photoresist was spin‐coated on a 4‐inch silicon wafer and the pattern containing all the geometric features of the chamber layer except for the T‐shaped posts was transferred onto SU‐8 through laser light exposure (Maskless Aligner MLA100, Heidelberg). Second, the layout containing all the chamber layer features was transferred to a layer of 100 µm‐high SU‐8, which had been spin‐coated on top of the previous one. Following exposure, silicon wafers were cured and developed according to the manufacturer's specification, thus obtaining master molds with features in relief. Single‐layer photolithography was instead used for the generation of the *valves* and *actuation layers*, obtaining 150 µm and 50 µm‐high SU‐8 features, respectively.

Replica molding of the micro‐structured master molds was then employed to fabricate PDMS devices. Initially, master mold surfaces were exposed to tri‐methyl‐chloro‐silane for 30 min at room temperature, to prevent PDMS from sticking to the wafer and facilitate its removal. Subsequently, a mixture of PDMS and curing agent at a 10:1 ratio was casted on the molds and polymerized at 65 °C for at least 3 h. In particular, a defined amount of PDMS was cast on the *chamber layer* master mold and on the *valve layer* master mold, to obtain controlled thicknesses (i.e., 300 µm and 400 µm, respectively) to guarantee proper valves and actuation functioning. After cross‐linking, PDMS stamps were peeled off from the silicon wafers, and an access port on the *actuation layer* was created using a 1.5 mm biopsy puncher. Thus, *actuation layer* and *valve layer* underwent a plasma treatment (i.e., 30 W for 50 s at 0.420 torr, Harrick Plasma Inc) and were brought into conformal contact to achieve irreversible bonding after 15 min at 65 °C. The assembly was then placed on top of the *culture chamber layer* through plasma bonding (Figure S1a). The assembly was finalized by punching circular access ports with biopsy punchers (5 mm for chamber reservoirs, 1 mm for gel injection ports). Finally, the microfluidic platform was completed by plasma bonding the whole assembly onto a glass slide. To produce microfluidic devices with functioning valves, this last step was performed under vacuum condition. In details, a Tygon tubes was inserted in the valves access port and connected to a vacuum chamber at ‐0.8 bar prior to bonding, thus guaranteeing valve opening during bonding procedure and avoiding permanent bonding of the separating walls on the glass slide.

### Calibration of the Valve Operating Pressure

A Tygon tube was plugged into the *valve layer* access port and connected to a mercury column with a pressure gauge. The *valve layer* was filled with a red dye, while the *chamber layer* was filled with PBS. Relative pressure in the valve chamber was stepwise decreased from 0 to −660 mmHg (step of 20 mmHg) and images were captured through an optical microscope (B120c, AmScope). As shown in Figure S1b, the valve appeared completely red in the rest position (i.e., at 0 mmHg), while decreasing pressure within the valve layer led to PDMS wall deflection toward the valve system, displacing the red color dye and restoring the wall to a transparent color at maximum opening. Images were then analyzed through ImageJ, to build a calibration curve. Specifically, images were converted into an 8‐bit format and five regions of interest (ROIs) corresponding to the PDMS walls beneath each of the five valves were chosen. Mean gray intensity (MGI) value was calculated in each ROI for each pressure level, as reported in the following equation:

(1) $$Normalized\;MGI_{ROI}=\frac{MGI_{ROI}-MGI_{ROI\;@\;atm\;pressure}}{MGI_{ROI\;@\;highest\;pressure}}\;$$

In details, background (i.e., mean gray intensity obtained at atmospheric pressure) was subtracted from measurements, and values were expressed as percentage of the mean gray intensity obtained at the highest pressure applied to normalize for light conditions. Measurements from the five ROIs in three different devices (n = 3) were averaged.

### Communication Valves Technical Validation

The entire device was primed with PBS, keeping *communication valves* open, to avoid air bubble formation. After valve closure, PBS was removed from the reservoirs of the medium channels of the *synovium* and *cartilage compartments*, that were subsequently filled with green and blue dyes, respectively. Finally, c*ommunication valves* were opened, and mixing of color dyes through the communication channels was observed through an inverted microscope.

### Evaluation of FITC‐Dextran Diffusion

The central channel of each culture compartment was injected with fibrin gel, formed by mixing fibrinogen (FB, Merck Aldrich) and thrombin (TH, Baxter TISSEEL) to achieve final concentrations of 10 mg mL^−1^ and 2.5 U mL^−1^, respectively. Medium channels were filled with PBS after gel cross‐linking (10 min at 37 °C), maintaining *communication valves* open. All the reservoirs were then filled with 60 µl PBS, except for the reservoirs of the outermost medium channel of the *synovium compartment*. At t = 0, the inlet and outlet reservoirs of the outermost medium channel of the *3D chamber* were filled with 60 µl of 40 kDa FITC‐dextran (1.5 mg mL^−1^ in PBS, Figure S1d). FITC‐dextran diffusion toward the *cartilage compartment* was monitored through an inverted fluorescence microscope (Olympus IX83): representative images were taken every 10 min for the first 110 min, and then every 30 min up to 620 min. Images were then processed through ImageJ software to compute variation of FITC‐dextran fluorescence intensity over time in the central channel of *cartilage compartment*. Specifically, each image was converted into an 8‐bit image and gray intensity values were computed inside three squared ROIs, identified along the gel channel of the *cartilage compartment* (white squares in Figure 1g). Gray values for each ROI were normalized against the maximum gray value of the picture. Normalized MGI was thus obtained averaging the values computed in the 5 ROIs of three different platforms.

### Cell Harvesting and Expansion

Human primary monocytes were isolated via density gradient separation with Ficoll (GE Healthcare) and magnetic cell separation from buffy coats from healthy donors purchased from the local blood bank (ASST Rodense).^[^ ^107^ ^]^ Briefly, blood diluted 1:1 with PBS was layered on top of Ficoll and centrifuged at 400 g for 30 min at room temperature. After centrifugation, peripheral blood mononuclear cells (PBMCs) were collected from the white ring, to proceed to magnetic cell separation using CD14^+^ Isolation Kit (Miltenyi Biotech) according to manufacturer's instructions. Freshly isolated monocytes (P0) from 3 donors were pooled, seeded at 4×10^5^ cells cm^−2^ in T25 culture flasks and cultured for five days in serum‐free X‐vivo 15 (Lonza) in a humidified incubator, supplemented with 20 ng mL^−1^ macrophage colony‐stimulating factor (MCSF, R&D Systems), aiming at inducing differentiation toward macrophages. After pre‐differentiation, suspended macrophages were collected, and adhered macrophages were detached by incubation at 37 °C for 5 min in Cell Dissociation Buffer (ThermoFisher). Harvested macrophages were used immediately after differentiation, without further passaging.

Human primary synovial fibroblasts (SFBs, male, 65 y.o., Lot 3343, Cell Applications) were thawed at P5 using complete culture medium, i.e., DMEM High Glucose (Gibco) supplemented with 1 mM Sodium Pyruvate (Gibco), 10 mM HEPES Buffer solution (Gibco), 100 U mL^−1^ penicillin, 100µg mL^−1^ streptomycin, 0.292 mg mL^−1^ L‐glutamine (Gibco), 10% Fetal Bovine Serum. SFBs were plated in culture flasks at a density of 4000 cells/cm^2^ and cultured in complete medium for seven days in a humidified incubator. Cells at P6 were then rinsed with PBS and harvested using 0.05% Trypsin – EDTA 0.02% (Gibco).

Primary healthy human articular chondrocytes (hACs) obtained from a 30‐year‐old male donor (donor 31 343, lot. 000 0604841, Lonza) were thawed at P4 and plated in culture flasks at a density of 4′444 cells/cm^2^ in complete culture medium, supplemented with 1 ng mL^−1^ TGF‐β1 and 5 ng mL^−1^ FGF‐2, and cultured in a humidified incubator for four days. hACs at P5 were then rinsed with PBS and collected using 0.05% Trypsin – EDTA 0.02% (Gibco).

### Generation of a Healthy Cartilage Model Inside the JoC Platform

After cell count and centrifuging, hACs were suspended in a fibrin gel (10 mg mL^−1^ FB, 2.5 U mL^−1^ TH) at a cell density of 50×10^6^ cells mL^−1^ and injected in the central channel of the *cartilage compartment* (Day_cart_ 0, Figure 2a). Cell‐laden hydrogel was allowed to cross‐link in a humidified incubator for 10 min, and medium channels were filled with chondrogenic medium, i.e., High Glucose DMEM with 10mM Hepes, 1 mM sodium pyruvate, 100 U mL^−1^ penicillin, 100 µg mL^−1^ streptomycin, 0.292 mg mL^−1^ L‐glutamine, 1% ITS+1 Liquid Media Supplement (Merck) and 125 mg mL^−1^ human serum albumin (HSA, Merck), supplemented with 10 ng mL^−1^ TGFβ3 (Peprotech), 10^−7^ M dexamethasone (Merck), 0.1 mM ascorbic acid (AA, Merck). Aminocaproic Acid (ACA, Merck) 2 mg mL^−1^ was added to the culture medium. The cells were cultured for 14 days, with the medium changed every other day, gradually decreasing the concentration of ACA (day 2: 1.6 mg mL^−1^, day 4: 1.2 mg mL^−1^, day 6 and thereafter: 0.8 mg mL^−1^), following a standardized procedure validated in the laboratory across different experimental settings to prevent excessive and premature degradation of the fibrin matrix.^[^ ^29^, ^108^, ^109^, ^110^ ^]^ At the end of the two weeks (Day_cart_ 14), samples were collected for RT‐PCR and immunofluorescence staining as described below, to assess cartilage maturation.

### Induction of an OA Phenotype on Cartilage Micro‐Tissues through Mechanical Overload

On Day_cart_ 14, the JoC platforms were connected to a compressed air source and a HPC of 30% was applied to the *cartilage compartment* using a pattern that resembles the daily walk (i.e., frequency of 1Hz, 2h stimulation – 4h rest – 2h stimulation – 16h rest). Control devices were cultured under static conditions. Culture medium was replaced with chondrogenic medium supplemented with 0.1 mM AA, which was changed every other day for one week. On Day_cart_ 21, samples were collected for RT‐qPCR and immunofluorescence staining, to assess the induction of OA traits.

### Generation of a Synovium Model Inside the JoC Platform

After cell counting and centrifuging, monocyte‐derived macrophages (MΦs) and SFBs were mixed in a 1:1 ratio and embedded in a fibrin‐collagen gel at a final cell density of 25×10^6^ cells mL^−1^ (12.5×10^6^ cells mL^−1^ MΦs, 12.5×10^6^ cells mL^−1^ SFBs). Briefly, human collagen type I (Corning) was mixed with PBS 10x and 2 mM HCl according to manufacturers’ specification, to reach a final concentration of 2 mg mL^−1^. Fibrin gel was prepared as described above, to obtain a final concentration of 20 mg mL^−1^ FB and 4 U mL^−1^ TH. Fibrin‐collagen gel was then prepared by mixing 50% of collagen and 50% of fibrin solutions, respectively. On Day_syn_ 0, cell‐laden hydrogel was injected in the central channel of the *synovial compartment* and allowed to cross‐link for 10 min in a humidified incubator (5% CO_2_, 37 °C). Lateral medium channels were then filled with serum‐free X‐vivo 15, supplemented with 20 ng mL^−1^ MCSF and 2 mg mL^−1^ ACA. MΦs and SFBs were cultured for up to 7 days. Culture medium was changed every other day, gradually decreasing ACA concentration (i.e., day 2: 1.6 mg mL^−1^, day 4: 1.2 mg mL^−1^, day 6: 0.8 mg mL^−1^). On Day_syn_ 0, Day_syn_ 3 and Day_syn_ 7, samples were collected for viability assay, RT‐qPCR and immunofluorescence staining.

### Induction of a Pro‐Inflammatory Phenotype on Synovial Micro‐Tissues through Biochemical Stimulation

MΦs and SFBs embedded in a fibrin‐collagen gel at a total cell density of 25×10^6^ cells mL^−1^ were seeded in the platforms on Day_syn_ 0, as described above. Control chips (“*Untreated*”) were cultured with X‐vivo 15 supplemented with 20 ng mL^−1^ MCSF and 2 mg mL^−1^ ACA, while stimulated samples (“*Inf*”) were cultured with serum‐free X‐vivo 15 supplemented with 20 ng mL^−1^ MCSF, 100 ng mL^−1^ TNFα, 100 ng mL^−1^ IFNγ and 2 mg mL^−1^ ACA. Culture medium was changed every other day, gradually decreasing ACA concentration. Pro‐inflammatory stimulation was maintained up to Day_syn_ 3, when culture medium was switched to X‐vivo 15 supplemented with 20 ng mL^−1^ MCSF. Synovial micro‐constructs were cultured up to Day_syn_ 7. On Day_syn_ 0, Day_syn_ 3 and Day_syn_ 7, samples were collected and analyzed by flow cytometry, RT‐qPCR and immunofluorescence staining.

### Co‐culture of 3D Cartilage and Synovium Tissue Within the JoC Platform

On Day_tot_ 0 (i.e., Day_cart_ 0), hACs were stained with Vybrant DyeCycle Green Stain (Invitrogen) according to manufacturer's protocol and were seeded in the *cartilage compartment* in fibrin gel at a density of 50×10^6^ cells mL^−1^ and allowed to mature inside the JoC platform for 14 days (i.e., Day_cart_ 14, Day_tot_ 14) in chondrogenic medium, supplemented with 10 ng mL^−1^ TGFβ3, 10^−7^ M DEX, 0.1 mM AA and 2 mg mL^−1^ ACA. Culture medium was changed every other day, gradually decreasing ACA concentration. On Day_tot_ 14 (i.e., Day_cart_ 14), culture medium was replaced with chondrogenic medium supplemented with 0.1 mM AA. On the same day (i.e., Day_syn_ 0), MΦs and SFBs were stained with Vybrant DyeCycle Green Stain and Vybrant DyeCycle Ruby Stain, respectively, and seeded in the *synovial compartment* in fibrin‐collagen gel at a density of 25×10^6^ cells mL^−1^, and cultured up to Day_tot_ 21 (i.e., Day_syn_ 7) in X‐vivo 15 supplemented with 20 ng mL^−1^ MCSF and 2 mg mL^−1^ ACA. Culture medium was changed every other day, gradually decreasing ACA concentration. *Communication valves* were open at Day_tot_ 17 (i.e., Day_cart_ 17, Day_syn_ 3). Brightfield and fluorescence images were taken at Day_tot_ 0, Day_tot_ 14, and Day_tot_ 21 to monitor micro‐tissues during the whole culture period. On Day_tot_ 21, synovial and cartilage micro‐tissues were collected for RT‐PCR. Devices containing synovium only and cartilage only were maintained in culture for 7 days and 21 days, respectively, and used as controls (namely *“Single culture”*). Specifically, control cartilage micro‐tissues were seeded in the platform on Day_tot_ 0 and cultured up to Day_tot_ 14 in chondrogenic medium supplemented with 10 ng mL^−1^ TGFβ3, 10–7 M DEX, 0.1 mM AA and ACA. From Day_tot_ 14 to Day_tot_ 17, culture medium was switched to chondrogenic medium supplemented with 0.1 mM AA. On the other hand, control synovial micro‐tissues were seeded in the platform on Day_tot_ 14 and cultured up to Day_tot_ 17 in X‐vivo 15, supplemented with 20 ng mL^−1^ MCSF and 2 mg mL^−1^ ACA. On Day_tot_ 17, culture medium in both cartilage and synovium controls was replaced by a mix of culture media containing 50% of X‐vivo 15 supplemented with 20 ng mL^−1^ MCSF and 1.4 mg mL^−1^ ACA, and 50% of chondrogenic medium supplemented with 0.1 mM AA. Culture medium was changed every other day up to Day_tot_ 21, gradually decreasing ACA concentration.

### Effect of OA‐Like Synovium Tissue on Healthy Cartilage

hACs were cultured in flasks, seeded in the *cartilage compartment* on Day_tot_ 0, and cultured up to Day_tot_ 14 in chondrogenic medium, supplemented with 10 ng mL^−1^ TGFβ3, 10^−7^ M DEX, 0.1 mM AA and 2 mg mL^−1^ ACA, as described above. On Day_tot_ 14, culture medium was replaced with chondrogenic medium supplemented with 0.1 mM AA. On the other hand, MΦs and SFBs were pre‐cultured in 2D, seeded in the *synovial compartment* on Day_tot_ 14, and biochemically stimulated to induce synovial inflammation, as previously optimized, while maintaining *communication valves* closed. Briefly, stimulated samples (“*Inf*”) were cultured with serum‐free X‐vivo 15 supplemented with 20 ng mL^−1^ MCSF, 100 ng mL^−1^ TNFα, 100 ng mL^−1^ IFNγ and 2 mg mL^−1^ ACA, while untreated samples (“*Untreated*”) were cultured with X‐vivo 15 supplemented with 20 ng mL^−1^ MCSF and 2 mg mL^−1^ ACA. Culture medium was changed every other day, gradually decreasing ACA concentration. On Day_tot_ 17, pro‐inflammatory stimulation was removed from *synovial compartment* and culture medium was replaced with X‐vivo 15, supplemented with 20 ng mL^−1^ MCSF. On the same day, *communication valves* were opened and the micro‐tissues were co‐cultured up to Day_tot_ 21, keeping the valves open for the whole culture period. On Day_tot_ 21, synovial samples were collected for RT‐PCR, while cartilage micro‐tissues were collected for RT‐PCR and immunostaining.

### Effect of OA‐Like Cartilage Tissue on Healthy Synovium

hACs seeded in the *cartilage compartment* in fibrin gel (Day_tot_ 0) and cultured up to Day_tot_ 14, as described above. On Day_tot_ 14, culture medium was replaced with chondrogenic medium supplemented with 0.1 mM AA, and a HPC of 30% was applied to the *cartilage compartment* up to Day_tot_ 21. On the other hand, MΦs and SFBs were seeded on Day_tot_ 14 in the *synovial compartment*, as previously described. Culture medium supplied to the *synovium compartment* was X‐vivo 15 supplemented with 20 ng mL^−1^ MCSF and 2 mg mL^−1^ ACA, whose concentration was gradually decreased during the culture period. From Day_tot_ 17 and Day_tot_ 21, *communication valves* were opened. On Day_tot_ 21, cartilage samples were collected for RT‐qPCR, while synovium micro‐tissues were collected for RT‐PCR and immunostaining.

### Viability Assay

On Day_syn_ 0, Day_syn_ 3, and Day_syn_ 7, a viability assessment was performed on MΦs and SFBs seeded in the chip using a Live/Dead assay (Merck). The devices were washed three times with PBS and subsequently incubated in the dark at 37 °C for 15 min with a solution containing calcein (2µM) and ethidium homodimer‐1 (4µM). After rinsing with PBS, fluorescent images of 3 chambers per condition were captured using an inverted microscope. To quantify cell viability, ImageJ software was employed. Three ROIs were selected for each device, and within each ROI the green labelled cells (indicating live cells) and the red cell nuclei (indicating dead cells) were counted. Cell viability values were thus obtained by normalizing the number of alive cells against the total number of cells. Mean and standard deviation values were finally calculated and plotted for each experimental condition.

### RT‐qPCR

Cells at day 0 (either Day_syn_ 0 or Day_cart_ 0) were collected in Trizol (Merck) directly after detachment. For other time‐points, medium channels of the JoC platform were washed with PBS, and samples were collected in Trizol and the cells were collected by carefully peeling off the culture layer from the glass slides. Specifically, a drop of Trizol was added to the construct, and the sample was then collected using a pipette. Total RNA extraction by Trizol, cDNA synthesis and real‐time reverse transcriptase‐polymerase chain reaction (RT‐PCR, QuantStudio 7 Flex Real‐Time PCR System, ThermoFisher) were then performed according to standard protocols to quantify expression levels of the following genes of interest (Applied Biosystems): COL1A1 (Hs00164004_m1), COL4A1 (Hs00266237_m1), PRG4 (Hs00981633_m1), PTPRC (CD45, Hs04189704_m1), CDH11 (Hs00901479_m1), CD80 (Hs01045161_m1), CD86 (Hs01567026_m1), CD163 (Hs00174705_m1), MRC1 (CD206, Hs07288635_g1), IL6 (Hs00985639_m1), CXCL8 (IL8, Hs00174103_m1), MMP1 (Hs00899658_m1), MMP9 (Hs00957562_m1), COL2A1 (Hs00264051_m1), ACAN (Hs00153936_m1), COL10A1 (Hs00166657_m1), MMP13 (Hs00233992_m1), PTGS2 (COX‐2, Hs00153133_m1). Glyceraldehyde 3‐phosphate dehydrogenase (GAPDH, Hs02758991_g1) housekeeping gene was used as reference. For each condition, n≥3 biologically independent samples were considered.

### Immunofluorescence

Before fixation, chambers were incubated for 4h at 37 ° C with appropriate culture medium supplemented with Brefeldin A (Abcam), to inhibit protein transport processes and increase intracellular protein staining signals. Medium channels were then washed with PBS and the microtissues were fixed in 4% paraformaldehyde (PFA) overnight at 4 °C. Subsequently, the devices were disassembled by removing the glass coverslip from the culture compartment, eventually exposing the microtissues. Cells were permeabilized with 0.5% Triton‐X (Merck) PBS solution for 10 min. A blocking solution (3% goat serum (Merck), 0.3% Tween‐20 (Merck) in PBS) was applied for 1 h at room temperature to block non‐specific bindings. For immunostainings of synovial micro‐tissues, FcR blocking reagent (1:100, Miltenyi Biotec) was supplemented together with the blocking solution. Samples were then incubated overnight at 4 °C with primary antibodies. Mouse anti‐human collagen type‐I (dilution 1:100, Santa Cruz Biotech, sc‐293182) and rabbit anti‐human lubricin (1:200, Invitrogen, PA3‐118) were used to evaluate synovium maturation at Day_syn_ 0, Day_syn_ 3 and Day_syn_ 7, as well as the effect of HPC cartilage on synovium on Day_tot_ 21. Rabbit anti‐human CD80 (1:1000, abcam, ab225674), anti‐human MMP1 (1:150, abcam, ab52631), anti‐human MMP9 (1:500, abcam, ab76003), as well as mouse anti‐human CD86 (1:100, Santa Cruz Biotech, sc‐19617) were used to assess synovial inflammation, either in synovial microtissues cultured individually or in contact with HPC cartilage. Rabbit anti‐human collagen type‐II (dilution 1:200, abcam, ab34712) and mouse anti‐human aggrecan (dilution 1:100, Santa Cruz Biotech, sc‐33695) were used to evaluate the maturation of cartilage micro‐constructs at Day_tot_ 14. Finally, rabbit anti‐human MMP13 (dilution 1:200, abcam, ab39012), mouse anti‐human aggrecan (dilution 1:100, Santa Cruz Biotech, sc‐33695) and mouse anti‐human SOX9 (dilution 1:100, Santa Cruz Biotech, sc‐166505) were employed to assess the effect of mechanical compression, as well as of synovial inflammation on cartilage micro‐tissues on Day_tot_ 21. After incubation with primary antibodies, samples were rinsed with blocking solution and, as appropriate, secondary antibodies labelled with Alexa Fluor 488, Alexa Fluor 546, and Alexa Fluor 647 (Invitrogen) were supplied at 1:200 dilution for 2 h at room temperature. Finally, samples were incubated for 30 min at RT with nuclear staining solution (DAPI, 300 nM, ThermoFisher). Representative images of three different regions for each microtissue were acquired using a confocal microscope (Leica) and analyzed using ImageJ software. Three microtissues for each condition were considered for the immunofluorescence analysis.

### Flow Cytometry

Medium channels were washed with PBS and synovial micro‐constructs were digested with a buffer containing 1 mg mL^−1^ Collagenase type‐I, 1 mg mL^−1^ Collagenase type‐II (Merck) and 200 U mL^−1^ Nattokinase in PBS, for 10 min at 37 °C. Collected samples were then centrifuged at 500g for 5 min. Subsequently, cell pellets were suspended in 250 µL MACS buffer (Miltenyi Biotec) and stained to assess the expression of surface markers using specific antibodies, according to manufacturers’ instructions. Before staining, cells were pre‐incubated with FcR blocking reagent (Miltenyi Biotec). The antibodies (Miltenyi Biotec) used for staining included anti‐human CD45 APC‐Vio 770 (130‐116‐159), anti‐human CD80 APC (130‐117‐831), and CD86 FITC (130‐116‐262). Specifically, CD45 was selected as marker to discriminate macrophages (i.e., CD45^+^) from synovial fibroblasts (i.e., CD45^−^), while all the other antibodies were selected as macrophage phenotypic markers. All antibodies were used at 1:50 dilution and all staining procedures were conducted at 4 °C for 20 min in the dark. Unstained cells served as negative controls for fluorescence. Data were acquired using a Cytoflex flow cytometer (Beckman Coulter Inc.). The flow cytometry analysis involved evaluating both the percentage of cells expressing each marker and the Mean Fluorescence Intensity (MFI) associated with the cell population for each specific marker.

### Bead‐Based Multiplexed ELISA

Multiplexed ELISA on chip supernatants were performed using custom kits of pre‐mixed antibody‐coated beads (Human Magnetic Luminex Assays, R&D Systems), which included the following analytes: CCL2, CCL3, CCL5, MMP1, IL6, IL8, IL10, ICAM‐1, Osteopontin. Briefly, based on manufacturer's instructions, 50 µl of media or kit standards were added to each well and incubated with a diluted microparticle cocktail for 2 h on a shaker at 800rpm. After washing the unbound soluble molecules, the biotin–antibody cocktail specific to the analytes of interest was added to each well for 1 h at room temperature. Wells were washed again, and conjugated streptavidin–phycoeriythrin was added for 30 min at room temperature. After washing, microparticles were resuspended in the provided washing buffer and read on a Bio‐Rad Bio‐Plex analyzer.

### Statistical Analysis

Statistical analysis was performed using GraphPad Prism software. Normally distributed data populations were assessed using Shapiro‐Wilk test. Two tailed Student's T‐test (normal distributions) and Mann‐Whitney test (non‐normal distributions) were used when comparing two populations. Multiple comparisons were realized using ordinary one‐way ANOVA. Statistical significance was indicated by ^*^ *p*<0.05, ^**^ *p*<0.01 and ^***^ *p*<0.001.

### Ethical Statement

The primary human monocytes/macrophages used in this study are obtained from commercial buffy coats purchased from a local blood bank. Buffy coats are provided in an anonymized form without providing donor gender, age, or any other relevant data. The buffy coats are used uniquely for monocyte isolation. The isolated cells are not used for any diagnostic and therapeutic purpose and no genetic investigation is conducted on cells. Based on all these considerations, ethics approval is not required for the isolation and use of these cells and blood donors are not required to provide any informed consent related to the use of the cells or to the publication of data derived from the use of these cells.

## Conflict of Interest

Marco Rasponi and Paola Occhetta share equities in BiomimX Srl.

## Author Contributions

S. L. and P. O. contributed equally to this to this work. P.O. and S.L. conceived the project. C.P. designed and fabricated the devices, performed the experiments, analyzed the readouts, worked on data visualization, and wrote the original draft. S.S. contributed to experimental design, cell isolation, cell expansion and experimental work. S.L. provided support in macrophage culture and marker analysis through flow cytometry. M.A.P. performed LumineX analyses. P.O., S.L., and M.R. contributed to the planning and supervision of the research activity and to interpreting the results. M.R. and M.M. supported project supervision. P.O. and M.R. managed the project. C.P. was primarily responsible for writing the manuscript, with all authors discussing the results, commenting on the manuscript, and contributing to its final version.

## Supporting information



Supporting Information

Supplemental Video 1

## Data Availability

The main data supporting the findings of this study are available within the paper and its Supplementary Information. All data generated for this study are available from the corresponding author upon reasonable request.
