# Supplementary material for: A Compartmentalized Joint‐on‐chip (JoC) Model to Unravel the Contribution of Cartilage and Synovium to Osteoarthritis Pathogenesis
Source: Adv Sci (Weinh). 2025 Sep 11;12(42):e00374. doi: 10.1002/advs.202500374 (PMC12622499; doi:10.1002/advs.202500374)
Supplement: Supplementary file 1 — Supporting Information [file ADVS-12-e00374-s001.pdf]

SUPPORTING INFORMATION

A compartmentalized Joint-on-Chip (JoC) model to unravel the contribution of cartilage and synovium to osteoarthritis pathogenesis

Cecilia Palma, Shima Salehi, Michela Anna Polidoro, Matteo Moretti, Marco Rasponi, Silvia Lopa†, Paola Occhetta†

†Equally contributing authors

C. Palma, M. Rasponi, P. Occhetta
Department of Electronics, Information and Bioengineering
Politecnico di Milano
Via Ponzio 34/5, Milan 20133, Italy
E-mail: marco.rasponi@polimi.it

S. Salehi, M. Moretti, S. Lopa
Cell and Tissue Engineering Laboratory
IRCCS Istituto Ortopedico Galeazzi
Via Cristina Belgioioso 173, Milan 20157, Italy

M. A. Polidoro
Hepatobiliary Immunopathology Laboratory
IRCCS Humanitas Research Hospital
Via Alessandro Manzoni, 56 Rozzano, Milan 20089, Italy

M. Moretti
Regenerative Medicine Division, Institute for Translational Research
Ente Ospedaliero Cantonale – Università della Svizzera Italiana
Via Chiesa 5, Bellinzona 6500, Switzerland

M. Moretti
Euler Institute, Faculty of Biomedical Sciences
Università della Svizzera Italiana (USI)
Via Buffi 13, Lugano 6900, Switzerland

P. Occhetta
BiomimX Srl
Viale Decumano 41, MIND – Milano Innovation District, Milan 20157, Italy

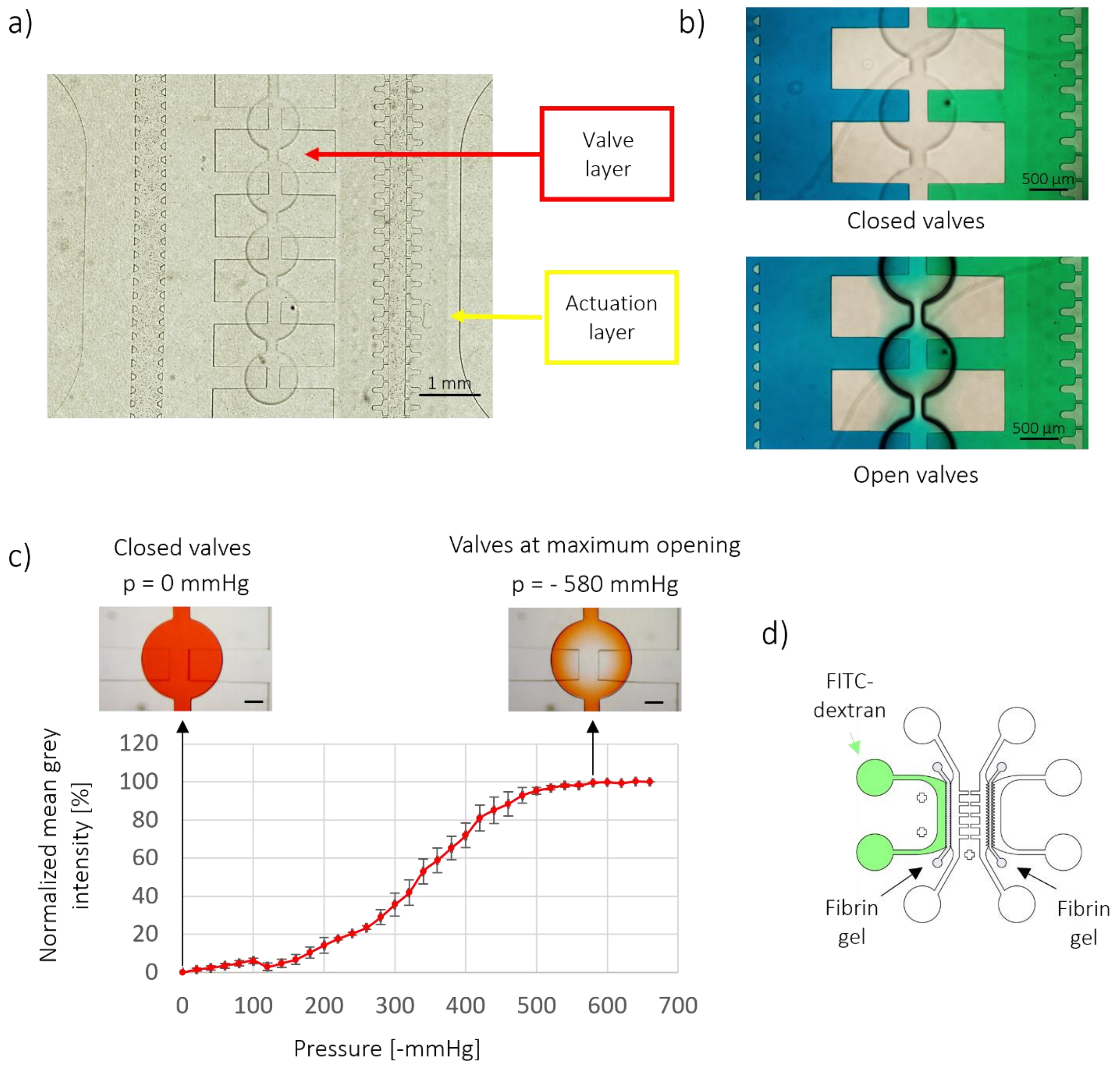

Figure S1. Device fabrication & Communication valves technical validation. *a) Brightfield image of an assembled device, showing actuation and valve layer aligned on top of the culture chamber layer. Scale bar 1 mm. b) Brightfield images showing synovial (filled with blue dye) and cartilage compartment (filled with green dye), that are independent at rest, i.e. with closed communication valves. Upon communication valves opening, color dyes start mixing. Scale bar 500µm. c) Calibration curve of valve opening pressure through measurement of grey intensity values in the central part of each valve during the application of decreasing values of pressure. Scale bar 200µm. d) Schematic representation of the experimental set-up used to evaluate 40 kDa FITC-dextran diffusion from the synovial to the cartilage compartment with open valves, simulating the diffusion of cytokines.*

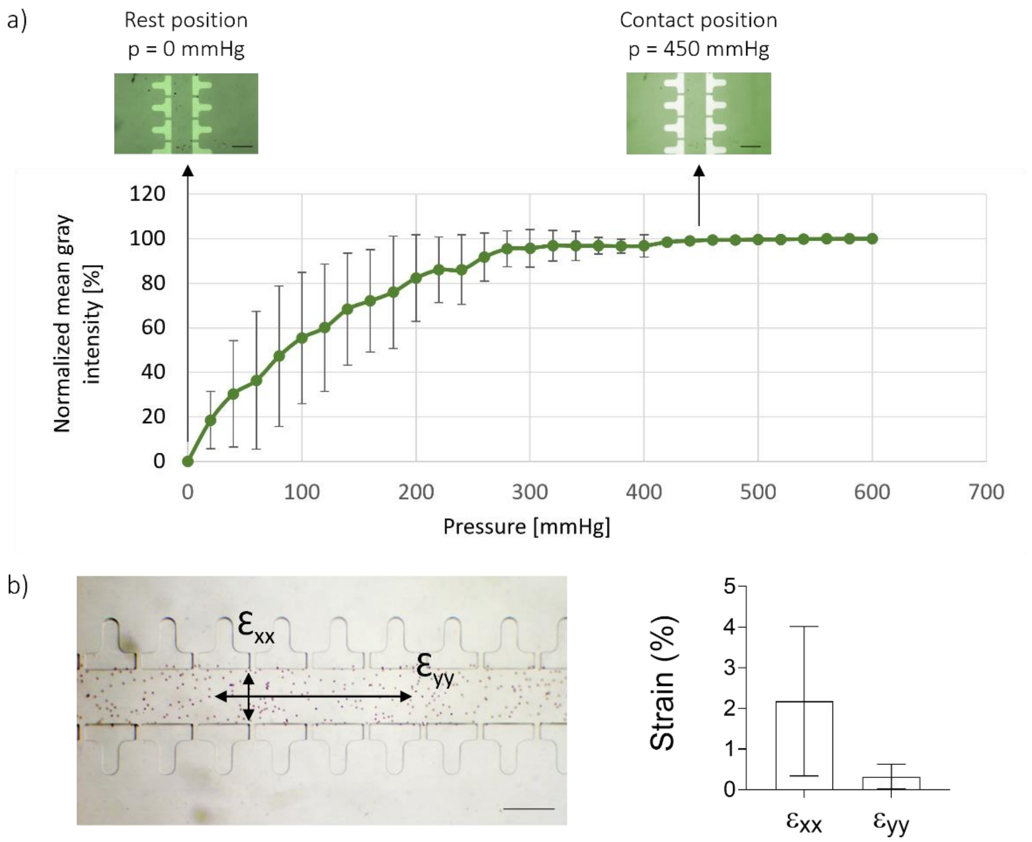

Figure S2. Mechanical characterization of the cartilage compartment. *a) Calibration of the actuation pressure - The actuation chamber was filled with PBS, and a green colored dye was injected in the cartilage compartment. At rest position (i.e., at 0 mmHg), the T-shaped posts appear light green because of the dye filling the gap between the glass slide and the overhanging posts. The actuation chamber was then connected to a pressurized air source through a Tygon tube inserted into the actuation access port, and the pressure was gradually increased with 20 mmHg increments, up to 600 mmHg. Images were taken through a stereomicroscope at every pressure increment: as the pressure increased, the posts' cross-section whitened due to the diminution of the gap between the posts and the glass slide, until they became completely white, meaning that the posts went in contact with the glass. Images were then processed through ImageJ, to measure mean gray values inside posts' cross-section, as described in Section 5.3. Measurements from nine pillars in three different devices were averaged for every pressure level. Results are expressed as mean ± SD (n=9). Normalized mean gray intensity (MGI) measured within the posts' cross-section allowed to identify plateau onset pressure. As shown from the graph, normalized MGI reached a plateau for pressure higher than 400 mmHg, meaning that at this pressure value the pillars abut on the glass slide (contact position). A pressure of 450 mmHg, i.e., 0.6 bar, was thus chosen for subsequent experiments. b) Quantification of mechanical deformation field along lateral (x) and longitudinal (y) direction - Polystyrene microbeads (10 µm diameter, Merck Aldrich) were incorporated into a fibrin hydrogel (FB 10 mg/ml, TH 2.5 U/ml). The microbead-laden gel was injected in the central channel of cartilage compartment and allowed to crosslink for 10 minutes in a humified incubator (37°C, 5% CO_2). After cross-linking, PBS was introduced into the medium channels and the device was connected to a pressurized air source, to apply a pressure of 0.6 bar in the actuation chamber. Images were captured using an optical microscope under both resting conditions (i.e., 0 bar) and compression condition. Scaler bar 300µm. ImageJ software was used to post-process the acquired pictures and measure microbeads' distances. Specifically, n=3 devices were considered and the mutual distance between 3 couple of beads per device was measured along x-direction (i.e., channel width, lateral direction) and y-direction (i.e., channel axial length, longitudinal direction) in rest and compression conditions. Lateral and longitudinal strains were computed as defined in the following equation:*

$$\varepsilon\varepsilon_{ii} = \frac{\Delta i - \Delta i_0}{\Delta i_0}$$

Where the index i indicates the direction (i.e., x or y), and Δi and Δi_0 correspond to the measured distances after compression and at rest, respectively. Mean value and standard deviation over the couple of beads were calculated. Data are plotted as mean ± SD. Strain values of 2.18 ± 1.84% and 0.32 ± 0.30% were obtained for lateral and longitudinal direction, respectively, in good accordance with experimental values measured in the previous version of the device (2.21% ± 2.20% and 0.59% ± 2.38%, respectively) [Occhetta et al. 2019] and confirming the achievement of a confined compression state within the cartilage compartment, with negligible transversal and longitudinal strains.

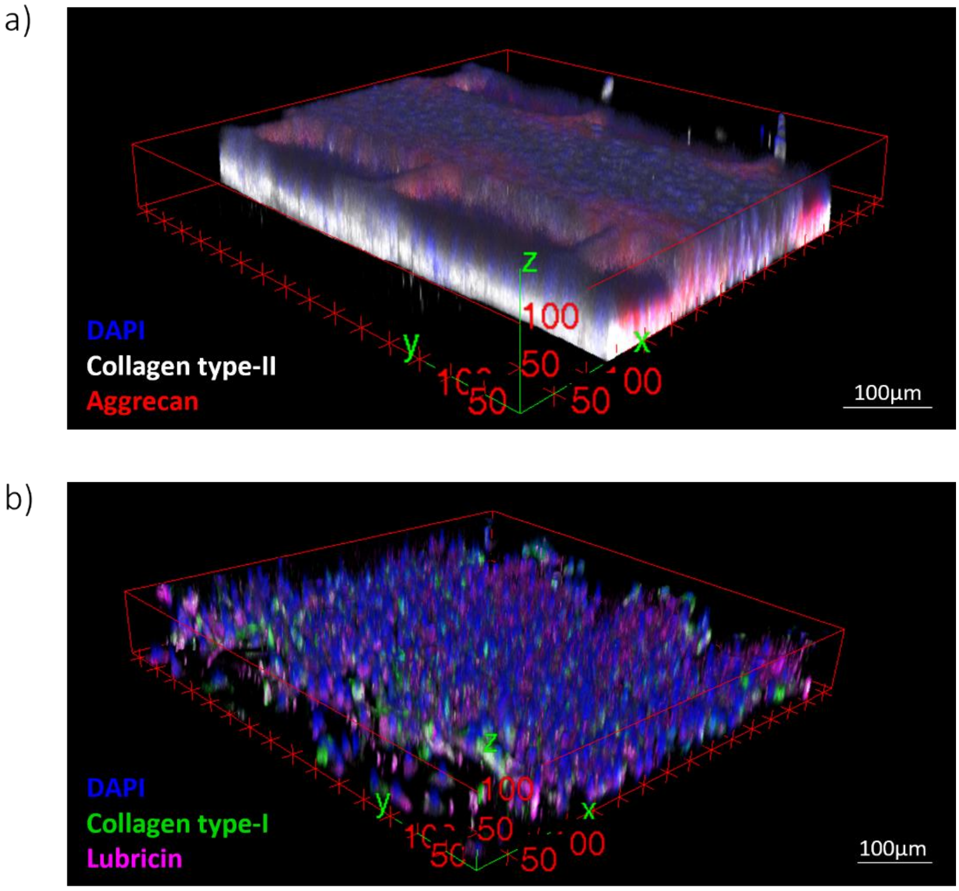

Figure S3. 3D reconstruction of micro-tissues inside the JoC platform *a) 3D reconstruction of cartilage micro-tissue on Day$_{cart}$ 14 (DAPI in blue, collagen type-II in grey, aggrecan in red). b) 3D reconstruction of synovium micro-tissues on Day$_{syn}$ 7 (DAPI in blue, collagen type-I in green, lubricin in magenta)*

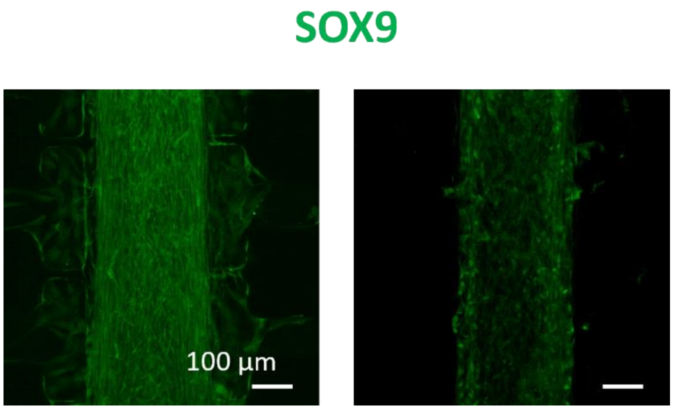

Figure S4: Induction of an OA phenotype on cartilage micro-tissues inside the JoC platform. *Confocal images showing SOX9 (green) inside the cartilage micro-tissues on Day$_{cart}$ 21, in static vs. HPC samples. Scale bar 100µm.*

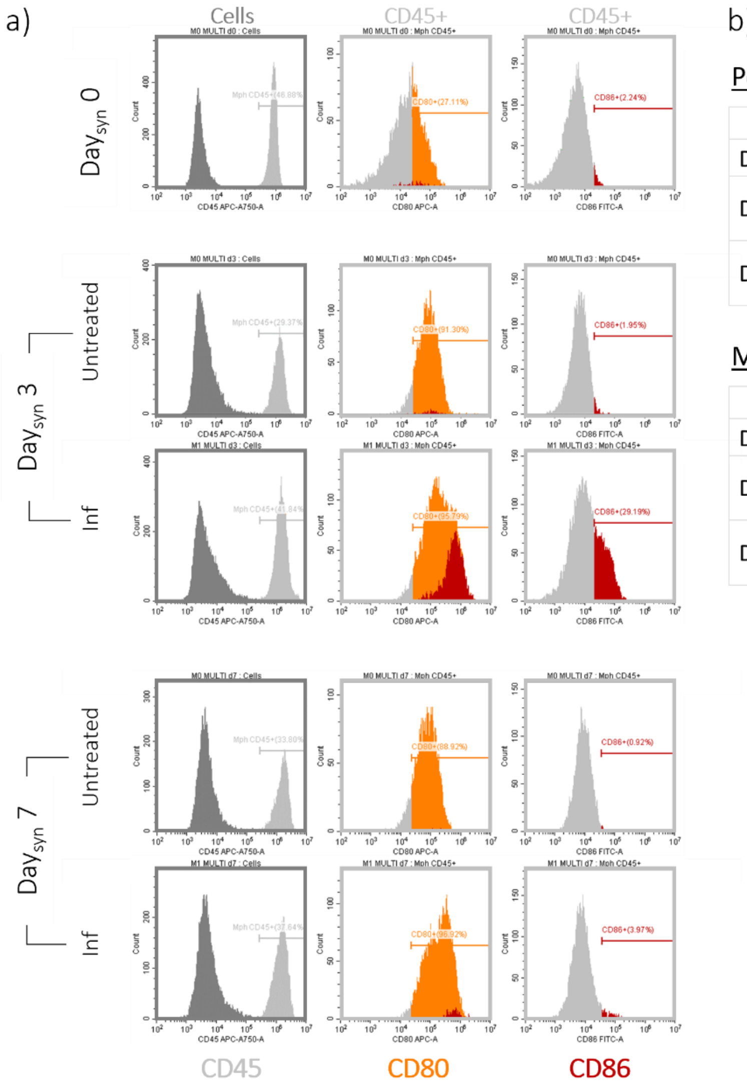

b)

Percentage of positive cells [%]

		CD80	CD86
Day$_{syn}$ 0	Untreated	27.11%	2.24%
Day$_{syn}$ 3	Untreated	91.30%	1.95%
	Inf	95.79%	29.19%
Day$_{syn}$ 7	Untreated	88.92%	0.92%
	Inf	96.92%	3.97%

Mean fluorescence intensity

		CD80	CD86
Day$_{syn}$ 0	Untreated	24010	5766.8
Day$_{syn}$ 3	Untreated	111040.6	7545.4
	Inf	320144.4	21420.8
Day$_{syn}$ 7	Untreated	93173.5	10296.7
	Inf	267137.5	11575.2

Figure S5. Induction of synovial inflammation in the JoC platform. *a) Flow cytometric analysis of CD45, CD80 and CD86, performed on synovial micro-tissues, at Day$_{syn}$ 0, Day$_{syn}$ 3 and at Day$_{syn}$ 7, comparing control ('Untreated') and stimulated ('Inf') samples. CD80 and CD86 were analyzed on CD45+ cells, i.e. macrophages. Histograms shows the number of events in function of fluorescence intensity for each marker, and the number of positive cells is reported in each graph. b) The tables report the number of positive cells and the mean fluorescence intensity (MFI), for each marker, time-point, and condition.*

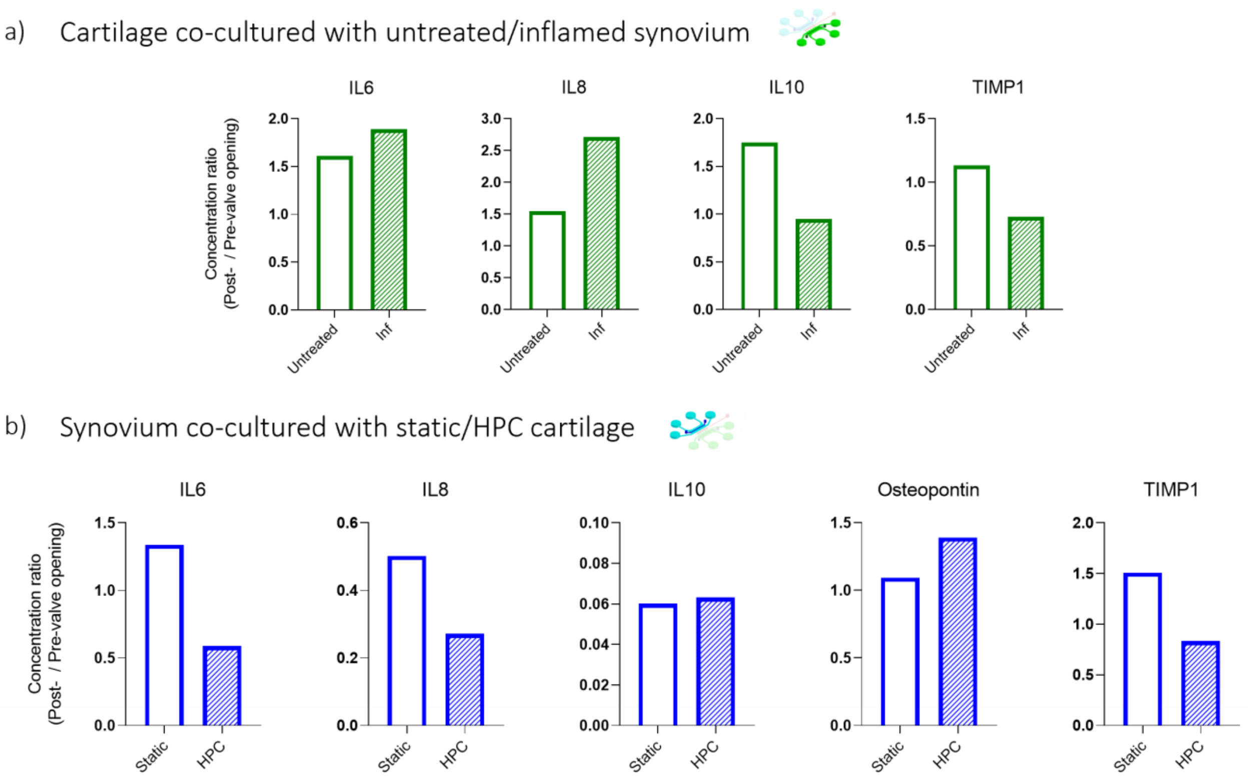

***Figure S6. Multiplexed ELISA (Luminex) on chip supernatant**. a) Ratio of soluble factors released from cartilage when co-cultured with synovium, presented as ratio between the amount released from Day$_{tot}$ 18 to Day$_{tot}$ 21 (i.e. after valve opening) and the amount released from Day$_{tot}$ 14 to Day$_{tot}$ 17 (i.e. before valve opening), thus reporting a relative increase/decrease upon co-culture. Samples co-cultured with untreated and inflamed synovium are shown. b) Ratio of soluble factors released from synovium when co-cultured with synovium, obtained presented as ratio between the amount released from Day$_{tot}$ 18 to Day$_{tot}$ 21 (i.e. after valve opening) and the amount released from Day$_{tot}$ 14 to Day$_{tot}$ 17 (i.e. before valve opening), thus reporting a relative increase/decrease upon co-culture. Samples co-cultured with static and hyperphysiologically compressed cartilage are shown.*

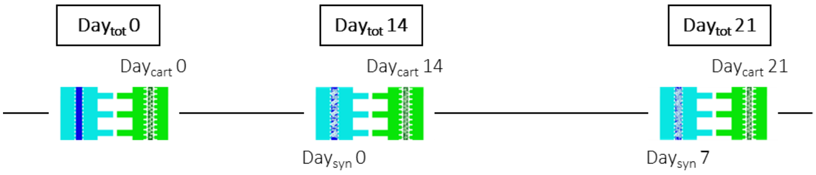

**Cartilage**		Day$_{cart}$ 14 vs Day$_{cart}$ 0	HPC vs Static (Single culture, Day$_{cart}$ 21)	Co-culture vs Single culture (Day$_{tot}$ 21)	Co-culture with Inf vs Untreated synovium (Day$_{tot}$ 21)	HPC vs Static in co-culture (Day$_{tot}$ 21)
Gene level	*PRG4*	↑*	x	x	x	x
	COL1A1	↑**	x	≈	≈	≈
	COL2A1	↑	≈	≈	↓	≈
	ACAN	↑***	≈	↑**	≈	↓
	COL10A1	x	x	↑**	≈	↓
	IL6	x	↑*	≈	↑**	↑
	IL8	x	↑	↑	↑	↑**
	MMP13	x	↑**	↑**	↑	↑*
Protein level	Collagen II	↑	x	x	x	x
	Aggrecan	↑	↓	x	↓	x
	MMP13	x	↑	x	↑	x

**Synovium**		Day$_{syn}$ 3 vs Day$_{syn}$ 0	Day$_{syn}$ 7 vs Day$_{syn}$ 0	Day$_{syn}$ 3 – Inf vs Untreated (Single culture)	Day$_{syn}$ 7 – Inf vs Untreated (Single culture)	Co-culture vs Single culture (Day$_{tot}$ 21)	Inf vs Untreated in co-culture (Day$_{tot}$ 21)	Co-culture with HPC vs Static Cartilage (Day$_{tot}$ 21)
Gene level	*CD45*	≈	≈	x	x	x	x	x
	PRG4	↑*	≈	↓**	≈	≈	≈	↑
	COL1A1	↑*	↑	↓	≈	≈	↓	↑
	COL4A1	↑*	↑**	≈	≈	x	x	x
	CDH11	x	x	≈	≈	x	x	x
	IL6	x	x	↑*	↑	↑*	↑	≈
	IL8	x	x	↑***	↑	↑	↑*	↓
	MMP1	x	x	↑**	↑	↑*	↑**	↓
	MMP9	x	x	≈	≈	≈	↑	≈
	CD80	x	x	↑**	↑**	x	x	x
	CD86	x	x	≈	≈	x	x	x
	CD163	x	x	↓*	↓	x	x	x
	CD206	x	x	↓	↓	x	x	x
Protein level	*Lubricin*	≈	↑	x	x	x	x	↑
	Collagen I	≈	↑	x	x	x	x	↑
	CD80	x	x	↑	↑	x	x	↑
	CD86	x	x	↑	↑ (IF)<br>≈ (FACS)	x	x	↑
	MMP1	x	x	x	x	x	x	≈
	MMP9	x	x	x	x	x	x	≈

Table S1. Summary of molecular and phenotypic changes across experimental conditions. *The table provides a comprehensive overview of gene- and protein-level alterations observed in healthy, mechanically stressed, or inflamed conditions for cartilage and synovium micro-tissues across the different experimental setups. Data were obtained through RT-qPCR, immunofluorescence, or flow cytometry analyses. Legend: ↑ Increased expression, ↓ Decreased expression, ≈ No changes. Statitiscal significance: *p<0.05, **p<0.01, ***p<0.001. Absence of annotation indicates non-significant changes. The presence of an "X" indicates that the specific marker was not assessed under the corresponding condition.*

Video S1 *- Visualization of the dynamic control of inter-compartmental communication through the valve system. Yellow and blue dyes were introduced in the two separate compartments of the JoC platform. With open communication valves, the dyes mix, indicating fluid exchange between compartments. When the valves are closed, the compartments are successfully re-isolated, demonstrating the reversible sealing capacity of the valve design.*
